# Characterization of Oligomers of Heterogeneous Size as Precursors of Amyloid Fibril Nucleation of an SH3 Domain: An Experimental Kinetics Study

**DOI:** 10.1371/journal.pone.0049690

**Published:** 2012-11-27

**Authors:** David Ruzafa, Bertrand Morel, Lorena Varela, Ana I. Azuaga, Francisco Conejero-Lara

**Affiliations:** Departamento de Química Física e Instituto de Biotecnología, Universidad de Granada, Granada, Spain; National Institute for Agricultural Research, France

## Abstract

Understanding the earliest molecular events during nucleation of the amyloid aggregation cascade is of fundamental significance to prevent amyloid related disorders. We report here an experimental kinetic analysis of the amyloid aggregation of the N47A mutant of the α-spectrin SH3 domain (N47A Spc-SH3) under mild acid conditions, where it is governed by rapid formation of amyloid nuclei. The initial rates of formation of amyloid structures, monitored by thioflavine T fluorescence at different protein concentrations, agree quantitatively with high-order kinetics, suggesting an oligomerization pre-equilibrium preceding the rate-limiting step of amyloid nucleation. The curves of native state depletion also follow high-order irreversible kinetics. The analysis is consistent with the existence of low-populated and heterogeneous oligomeric precursors of fibrillation that form by association of partially unfolded protein monomers. An increase in NaCl concentration accelerates fibrillation but reduces the apparent order of the nucleation kinetics; and a double mutant (K43A, N47A) Spc-SH3 domain, largely unfolded under native conditions and prone to oligomerize, fibrillates with apparent first order kinetics. On the light of these observations, we propose a simple kinetic model for the nucleation event, in which the monomer conformational unfolding and the oligomerization of an amyloidogenic intermediate are rapidly pre-equilibrated. A conformational change of the polypeptide chains within any of the oligomers, irrespective of their size, is the rate-limiting step leading to the amyloid nuclei. This model is able to explain quantitatively the initial rates of aggregation and the observed variations in the apparent order of the kinetics and, more importantly, provides crucial thermodynamic magnitudes of the processes preceding the nucleation. This kinetic approach is simple to use and may be of general applicability to characterize the amyloidogenic intermediates and oligomeric precursors of other disease-related proteins.

## Introduction

Protein amyloid aggregation is intimately associated with a wide range of disorders, among which there are some devastating human diseases such as Alzheimer's, Huntington's, Parkinson's, prion diseases or type II diabetes [1]. Protein aggregation is also a general problem in biotechnological applications of proteins and in some cases plays a biologically relevant role, such as for instance in the polymerization of actin [2]. For these reasons, experimental kinetics studies of protein aggregation have been of general interest since several decades ago [3]. Quantitative aggregation kinetics analysis can help to infer the detailed mechanism and provide insight into the identity and properties of key intermediates in the aggregation pathway. Understanding the mechanisms of protein aggregation is a key step to control protein aggregation and to delineate effective therapeutic strategies for protein aggregation disorders.

An increasingly large body of experimental and theoretical examples of protein aggregation kinetic studies can be found in the recently reviewed literature [4], [5]. Theoretical models usually treat protein aggregation as a multi-stage process, in which a number of consecutive and/or parallel stages occur to convert the soluble protein monomer to large amyloid aggregates [4]. These stages may include partial unfolding or refolding, oligomerization, conformational conversion, elongation, condensation, fragmentation, etc. The broad diversity of proposed models of aggregation can be understood in terms of a predominance of one or more of these stages in the overall aggregation kinetics, which results in a variety of kinetic behaviors, depending on the protein and/or the experimental conditions studied in each case [6].

Almost invariably, the formation of oligomeric species, normally soluble and sometimes structurally unstable or metastable, is a key step in the nucleation of amyloid structures. For example, the 40- or 42-residue forms of amyloid-beta peptide (Abeta) give rise to different oligomeric species with a relatively disordered structure in rapid equilibrium with the monomeric forms [7], [8]. Likewise, similar oligomers featuring dynamic structures have been identified during amyloid fibril formation of the yeast prion Sup35p, phosphoglycerate kinase or the SH3 domain of the PI3 kinase [9]–[11]. Nucleation is then completed by structural conversion of these intermediate species into small amyloid-like structures that act subsequently as templates triggering rapid growth of fibrils. Sometimes, native or native-like structures are forming the oligomeric aggregates and then conversion to amyloid structures takes place within the aggregates [12], [13].

The investigation of the mechanism of formation of soluble oligomers during the initial stages of aggregation is of vital importance because these species appear to constitute the main toxic agents in neurodegenerative diseases [14]. Supporting this hypothesis, a significant correlation was found between the levels of soluble Abeta peptide, including its oligomeric forms, and the degree of synaptic alteration, neurodegeneration and cognitive decline in Alzheimer's disease patients, whereas a similar correlation was not observed for the insoluble deposits [15]. Also for α-synuclein, increasing evidence suggests that non-fibrillar dimers or oligomers play a major role in the progress of Parkinson's disease [16]. Similarly, it has been shown with neuronal cell cultures that there is less cell death in the presence of large aggregates of poly-Q-rich Huntingtin than when the soluble fraction is present [17]. Certain non-fibrillar aggregates of transthyretin have also been shown to be toxic to neuronal cells under the conditions where the native tetramers and the mature fibrils have no significant toxicity [18].

The general toxic nature of prefibrillar aggregates has been further underlined by the finding that oligomeric species of proteins unrelated to disease, such as HypF-N from E. coli, the SH3 domain of PI3 kinase or equine lysozyme, are highly toxic in fibroblast and neuron cultures, whereas the amyloid fibrils of the same proteins show low toxicity [19], [20]. These and many other studies have emphasized on the importance of using well-characterized model proteins, even unrelated to disease, to investigate the mechanisms of amyloid aggregation.

In previous work, we have reported that the wild type form and several mutants of the SH3 domain of α-spectrin (Spc-SH3) are rapidly converted to amyloid fibrils at mild acid pH [21], [22]. We found that the rate of nucleation is strongly affected by sequence mutations and experimental conditions, in particular temperature, pH and the concentrations of protein and salt, resulting in considerable changes in the governing kinetics and also in the morphological properties of the finally assembled fibrils [23]. For instance, at low salt concentration the aggregation shows a considerable lag phase, as usually observed for nucleation-dependent polymerization, resulting in well-ordered straight and twisted amyloid fibrils. An increase in salt concentration removes the lag phase due to an increase in the rate of nucleation, which is no longer rate-limiting in the formation of fibrils. Under these conditions, a higher accumulation of amyloid nuclei results in curly and relatively disordered fibrils. Salt ions appear to act by lowering the apparent activation energy of fibril nucleation [23]. These results implied that the molecular events taking place prior to and during the nucleation of amyloid structures determine the fate of the protein chain along the subsequent aggregation cascade. Therefore, a more detailed knowledge of the molecular mechanism of nucleation could give additional insight upon the physicochemical factors governing the process and offer more opportunities to its manipulation and control.

In this work we analyzed quantitatively the kinetics of formation of amyloid nuclei of the N47A Spc-SH3 mutant using a range of biophysical techniques. Although most amyloid fibrillation studies with both disease-related and unrelated proteins have observed significant lag phases in the kinetics, typical of nucleation-dependent processes, during these lag phases the formation of amyloid nuclei is a rare process, difficult to observe experimentally, and the aggregation kinetics are mainly governed by the elongation of few preformed nuclei accompanied by other secondary processes. For this reason, we decided to work under conditions of fast nucleation, i.e., in the absence of a lag phase, so that amyloid nuclei are abundantly formed and the observed kinetics are mainly representative of the nucleation process itself, whereas subsequent processes such as fibril elongation or fragmentation can be neglected during the initial stages of the kinetics. Under the selected conditions the nucleation kinetics can be quantitatively described as an irreversible high-order reaction, suggesting that nucleation of amyloid structures is preceded by formation of oligomeric precursors in rapid equilibrium with the monomer. Using a battery of biophysical methods we showed that these oligomers occur at very low population, have a heterogeneous size and form by rapid association of partially unfolded protein monomers. We proposed a mathematical kinetic model in which stable amyloid nuclei form by a conformational change of the protein within the oligomers. The model explained very well the observed initial rates of aggregation and allowed us to derive thermodynamic magnitudes characterizing the amyloidogenic intermediate and its oligomerization, as well as the rate constants of conformational change that forms the amyloid nuclei.

## Materials and Methods

### Protein sample preparation

The Spc-SH3 domain mutants studied were overexpressed in *E. coli* and purified as described elsewhere [24]. ^15^N-labelled protein was produced in *E. coli* cultures using M9 minimal media with ^15^N-ammonium sulfate as the sole nitrogen source. For aggregation experiments the lyophilized protein was dissolved, unless stated, in the appropriate buffer at 4°C, centrifuged for 2 minutes and filtered through a 0.2 µm filter. Protein concentration was determined by measurement of absorbance at 280 nm using an extinction coefficient of 15220 M^−1^ cm^−1^.

### ThT and ANS fluorescence

Thioflavine T (ThT) or 1-Anilino-8-naphthalene sulfonate (ANS) fluorescence was continuously monitored during amyloid fibril formation at 37°C in a Varian Cary Eclipse spectrofluorimeter (Agilent Technologies, Santa Clara, CA) equipped with a Peltier-controlled thermostatic cell holder. To start aggregation, a freshly prepared protein solution was mixed with a concentrated stock solution of each dye previously prepared in the same buffer (100 mM glycine, pH 3.2, 100 mM NaCl). The sample was then placed into a fluorescence cuvette, which was previously thermostatized at 37°C, and covered with mineral oil to avoid evaporation. Fluorescence intensity of ThT at 10 µM was monitored at 485 nm using an excitation wavelength of 440 nm and the ANS fluorescence was measured at 470 nm with excitation at 370 nm using various ANS concentrations. Previous control experiments allowed us to establish the appropriate concentration of each dye for the experiments. A linear relationship between the ThT fluorescence and the mass amount of amyloid fibrils was established using preformed amyloid fibrils (Figure S1).

Initial rates of growth of amyloid structure were calculated by fitting the initial region of the ThT kinetics curves, corresponding to an approximate signal growth of less than 50% of the maximum, using a double exponential function:
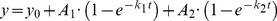
(1)


The initial slope is then calculated from the resulting fitting parameters as:
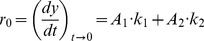
(2)


This operational extrapolation procedure allowed us an accurate determination of the initial slopes of the ThT curves and avoided the assumption of any time interval in the kinetics to define an initial aggregation rate.

### Circular dichroism (CD)

CD measurements were made in a Jasco J-715 spectropolarimeter (Tokyo, Japan) equipped with a thermostatic cell holder. Near UV CD spectra of the native N47A Spc-SH3 domain and the preformed amyloid fibrils were acquired at 4°C between 260 nm and 320 nm at a protein concentration of 8 mg mL^−1^ in a 1 mm path length cuvette (Figure S2). This short path length was necessary to avoid instrument saturation due to the high protein concentration of the samples. Far-UV CD measurements with the K43A-N47A double mutant were done at a protein concentration of 0.2 mg mL^−1^using a 1 mm path length cuvette. For kinetic experiments monitored by near-UV CD, a fresh protein sample was placed into a 1 or 2 mm cuvette, depending on the protein concentration of the experiment. The CD signal at 295 nm was then monitored at 37°C during the time course of aggregation. Data were averaged for 20 s using a band width of 1 nm and a response time of 4 s.

### Nuclear Magnetic Resonance

To follow the amyloid aggregation by two-dimensional NMR in real time, 4 mg of ^15^N-labelled protein was dissolved in 500 µL of cold buffer (100 mM d_6_-Gly, 100 mM NaCl, pH* 3.2) containing 10% D_2_O and placed into the NMR tube. The final protein concentration was 1.1 mM (8 mg mL^−1^). The temperature in the NMR probe was set to 37°C and, immediately after temperature equilibration, a series of ^1^H-^15^N HSQC two-dimensional spectra were acquired during the time course of aggregation in a 600 MHz Varian NMRsystem spectrometer (Agilent Technologies, Santa Clara, CA). Each spectrum consisted of 512×32 complex points recorded using a relaxation delay of 1 s and resulting in a total experiment time of 2 minutes and 28 seconds. The spectral widths were 12.04 ppm for ^1^H and 30.00 ppm for ^15^N. Spectra were processed using NMRPipe [25]. The assignment of the ^1^H-^15^N crosspeaks of the HSQC spectrum was performed using as a reference the previously published assignment of the WT Spc-SH3 domain [26]. To obtain the aggregation kinetics, the signal intensities were evaluated using NMRview [27] and represented versus the incubation time.

### Dynamic light scattering (DLS)

Aggregation was monitored at 37°C by DLS in a DynaPro MS-X instrument (Wyatt, Santa Barbara, CA, USA) using a thermostatized 30 µL quartz cuvette. The protein solution and the buffer were centrifuged and filtered through a 0.02 µm filters before the measurements. During the time course of the aggregation the DLS data were acquired every 45 seconds until saturation of the signal. The laser power was adjusted to avoid early saturation. Dynamics v.6 software was used in data collection and processing of the correlation function to finally obtain the particle size distributions during the course of aggregation.

### Attenuated total reflectance Fourier-transform infrared (ATR-FTIR) spectroscopy

Infrared spectra were recorded from 4000 cm^−1^ to 900 cm^−1^ on a Bruker IFS-66 FTIR spectrophotometer (Brucker, Ettlingen, Germany) equipped with a BioATR-II cell. For each sample, 128 interferograms were coadded and Fourier transformed with a zero filling factor of 4 to yield spectra with a nominal resolution of 2 cm^−1^. The sample temperature was controlled and set to 37°C by means of a thermostatic cell jacket. FTIR measurements were taken at 37°C every 90 seconds for the first 120 minutes of incubation and thereafter every 30 minutes. Buffer spectra were recorded under identical conditions and subtracted from the spectra of the protein sample (Figure S3). Spectral contributions from residual water vapor were reduced using the atmospheric compensation filter built in the Bruker OPUS software. Difference FTIR spectra were then calculated by subtracting the first measurement from all subsequent spectra. The change in the area of the difference band (from 1635 cm^−1^ to 1591 cm^−1^) was calculated and plotted versus time.

### Differential scanning calorimetry

The thermal unfolding of the K43A-N47A Spc-SH3 double mutant was monitored in a Auto-Cap VP-DSC instrument (Microcal, Northampton, MA) at a scan rate of 1.5°C min^−1^ and a protein concentration of about 1 mg mL^−1^. The molar partial heat capacity curve (C_p_) was calculated from the DSC data and analyzed using Origin 6.1 (OriginLab, Northampton, MA) according to the two-state unfolding model.

### Size-exclusion chromatography

Size-exclusion chromatography (SEC) experiments were performed at 37°C using a thermostatized Superdex 75 HR 10/30 column connected to an ÄKTA prime plus FPLC chromatograph (GE Healthcare, Tokyo). N47A Spc-SH3 samples were prepared in the aggregation buffer at the appropriate concentration and were incubated at 37°C in a water bath during different times. The same buffer was used for the elution at a flow rate of 1 mL min^−1^. A volume of 100 µL was loaded onto the column. The elution profiles were recorded by monitoring the absorbance at 280 nm. The column was previously calibrated using protein standards.

## Results

### Kinetics of formation of amyloid structure observed by thioflavine T fluorescence

**Figure 1:**
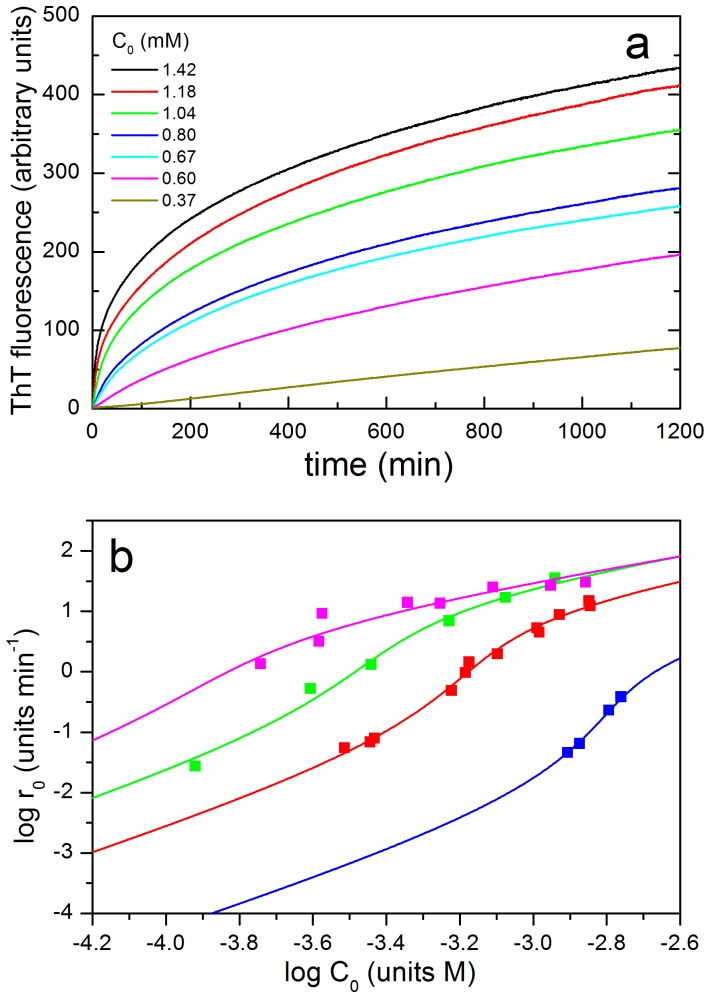
Kinetics of formation of amyloid fibrils followed by ThT fluorescence at 37°C. A: Kinetic curves of amyloid fibrillation of N47A Spc-SH3 in the presence of 100 mM NaCl at different initial protein concentrations as indicated. B: Double logarithmic plot of the initial rates of amyloid fibrillation of N47A Spc-SH3 versus the initial protein concentration in presence of different NaCl concentrations: 50 mM (blue); 100 mM (red) and 200 mM (green). The initial rates of the double mutant K43A-N47A Spc-SH3 in the presence of 100 mM NaCl are shown in magenta. Symbols correspond to the experimental rates and continuous lines represent the best fits according to equations 4 to 8 of the model as described in the text.

The kinetics of growth of amyloid fibrils of N47A Spc-SH3 was followed at 37°C at several protein concentrations by continuously monitoring ThT fluorescence at pH 3.2 in 100 mM glycine buffer, 100 mM NaCl. Under these experimental conditions, the development of amyloid structures takes place very rapidly without any significant lag phase, indicating a fast formation of amyloid nuclei (Figure 1A). No stirring or shaking of the samples was necessary to obtain rapid and reliable aggregation kinetics and we observed no significant sedimentation of the aggregates during the length of our experiments. This allowed us monitoring directly the aggregation kinetics using a variety of biophysical techniques, avoiding irreproducibility arising from the control of stirring.

The appearance of a significant amount of fibrils visible by electron microscopy is delayed under these conditions for at least 30 min (see Figure 10 in ref. [21]). Therefore, initial growth of ThT fluorescence could be attributed to the formation of small amyloid nuclei. The aggregation process does not follow first-order kinetics and its rate is strongly dependent of the initial concentration of protein. In addition, the ThT signal does not reach a plateau within the time of the experiments. Initial rates of formation of amyloid nuclei were estimated from initial slopes of the kinetic traces as described in Material and Methods. Using a double logarithmic plot, we found that the initial rates scale approximately with the fourth power of the initial protein concentration, indicating a high order for the nucleation of amyloid structure (Figure 1B, Table 1). Nevertheless, there is a slight but significant sigmoidal shape in this plot.

**Table 1:** Kinetic analysis of the apparent order for irreversible aggregation of Spc-SH3 using different biophysical techniques and methods of analysis.

Method	Type of analysis	Apparent order	log k (M^1−n^ min^−1^)	Protein concentration (mM)
ThT fluorescence	Initial rates^a^	3.80 ± 0.13	-	0.31–1.44
NMR	Non-linear fitting^b^	4.2 ± 0.3	9.2 ± 0.6	1.1
Near-UV CD	Initial rates^a^	6.1 ± 0.5	12.9 ± 1.4	0.84–1.6
	Non-linear fitting^b^	4.57 ± 0.07	9.2 ± 0.3	1.6
		4.73 ± 0.08	9.7 ± 0.3	1.4
		4.52 ± 0.08	8.9 ± 0.2	1.18
		4.62 ± 0.06	9.6 ± 0.2	1.14
		4.28 ± 0.06	7.9 ± 0.3	0.95
		4.0 ± 0.1	6.9 ± 0.3	0.84
ANS fluorescence	Initial rates^a^	3.1 ± 0.5	-	0.14–1.17
Far-UV CD	Initial rates^a^	1.5 ± 0.3	-	0.36–1.11

a Initial rates of signal growth were plotted versus the initial protein concentration in a double logarithmic plot. The apparent order is derived from the slope in a linear fit to this representation.

b The normalized signal intensity decay was fitted to the integrated equation of an irreversible n-order kinetics.

### Kinetics of depletion of native protein

**Figure 2:**
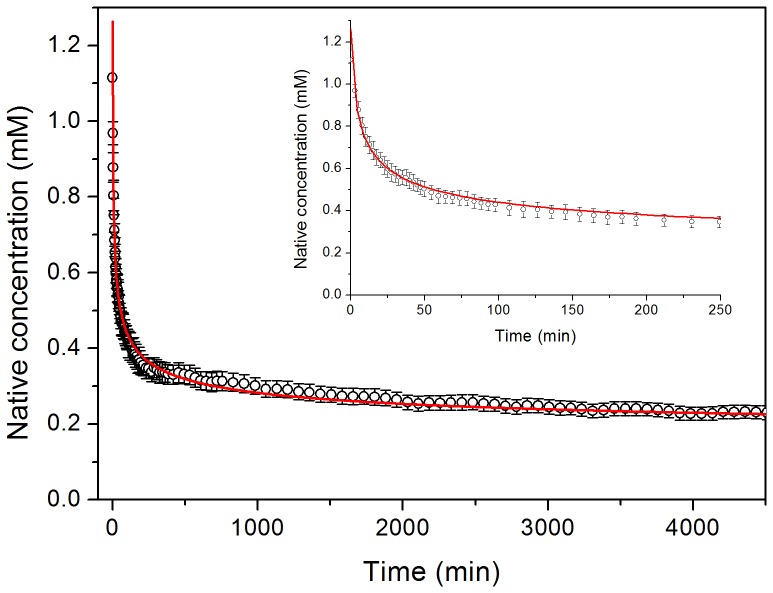
Kinetics of native-state depletion monitored by NMR. Averaged and normalized intensities of the cross peaks of all residues of N7A Spc-SH3 in a series of ^15^N-^1^H-HSQC NMR spectra recorded during the course of aggregation at 37°C using an initial protein concentration of 1.1 mM. The red continuous line represents the best fit using the integrated equation of an n-order irreversible kinetics. The inset shows an expansion of the early times of the kinetics.

We monitored the aggregation under the same experimental conditions by acquiring a series of ^15^N-^1^H HSQC NMR experiments during the course of the fibrillation process at 37°C using a 1.1 mM (8 mg mL^−1^) sample of ^15^N-labelled protein. This experiment was aimed at the detection of any early soluble intermediates of the protein, which may become populated prior to the formation of the fibrils. All spectra looked, however, identical to that of the native protein except for a rapid and continuous decrease in intensity (Figure S4). No significant shifts of the HSQC signals were observed during the whole process and the intensity decays of all the signals are virtually identical. These observations may suggest that the native protein is converted to large aggregates without accumulation of any intermediate visible by NMR. It is possible that interconversion of intermediates taking place in the NMR time scale could result in extreme line broadening, which renders invisible the signals. A plot of the normalized and averaged intensities of all the native HSQC peaks does not follow a single exponential decay (Figure 2). The kinetics could instead be very well fitted using the integrated equation of an n-order irreversible kinetics:
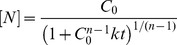
(3)


Where n is the apparent order of the reaction, C_0_ is the initial protein concentration, [N] is the concentration of native protein at time t and k is an apparent n-order rate constant. This fitting resulted in an apparent order of 4.2 ± 0.3 (Table 1).

**Figure 3:**
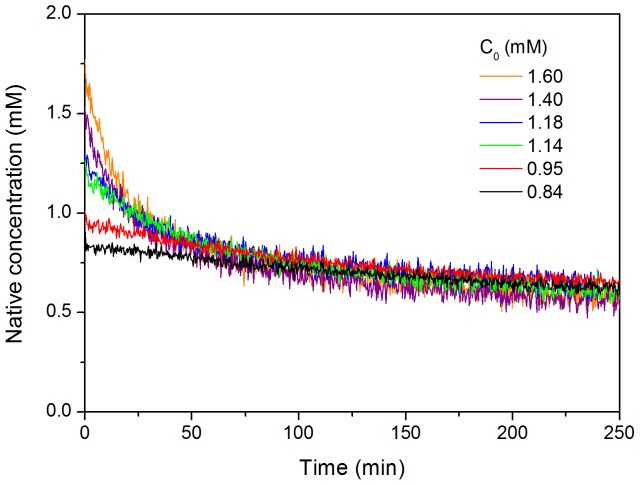
Kinetics of native state depletion monitored by near-UV CD. Different initial protein concentrations of N47A Spc-SH3 were analyzed as indicated. The intensity of the native CD signal at 295 nm was monitored as a function of time and normalized using the values of initial protein concentration.

The rate of disappearance of the native protein during the early stages of fibrillation was also monitored by near-UV CD at several protein concentrations ranging between 0.84 mM and 1.6 mM (Figure 3). In these experiments we took advantage of the absence of CD signal at 295 nm in the near-UV CD spectrum of the amyloid fibrils and we could assume therefore that the CD signal intensity at this wavelength is proportional to the concentration of the native protein monomer during the aggregation process. The kinetic curves are strongly concentration-dependent and in agreement with a high-order process. When normalized by the protein concentration, as expected for this type of kinetics, the curves converge approximately to a common trend. Global fitting of all the kinetic curves using equation 3 did not give fully satisfactory results especially for the curves at the lowest protein concentrations. Individual fittings yielded apparent orders ranging between 4 and 4.7 and relatively similar values for the apparent rate constants (Table 1). The initial rates of native state depletion, estimated by extrapolation of the slopes of the kinetics, are also strongly dependent on protein concentration. Double logarithmic plot of the initial rates versus the protein concentration provides an apparent order of about 6.

These results clearly demonstrate that during the early stages of aggregation, both the depletion of native protein and the growth of amyloid structures follow high-order irreversible kinetics. A simple interpretation of this result would suggest that the precursors of the rate-limiting step for amyloid nucleation are oligomers of about 4 to 6 protein molecules. Formation of these oligomeric precursor species from the native state of the protein seems to be therefore a necessary step preceding the nucleation process. Furthermore, this oligomerization must be sufficiently fast under these conditions to avoid a lag phase in the fibrillation.

### Formation of β-sheet structure

**Figure 4:**
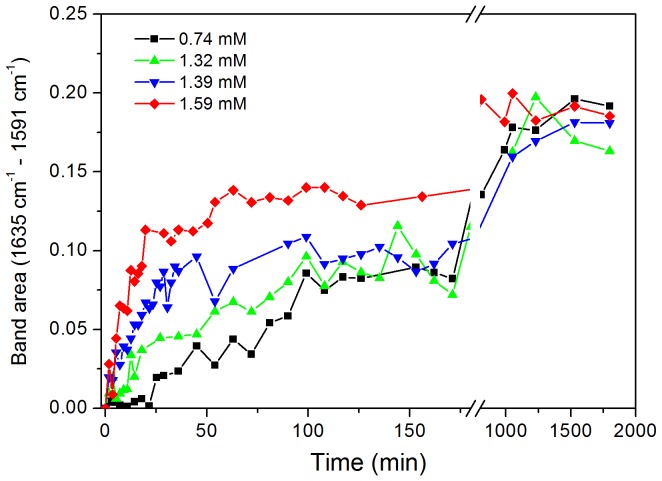
Kinetics of formation of beta-sheet structure during the course of aggregation. Aggregation of N47A Spc-SH3 was monitored by infrared spectroscopy at different initial protein concentrations as indicated in the figure. The data correspond to the area of the spectral band between 1591 cm^−1^ and 1635 cm^−1^ in the difference spectra obtained by subtracting initial spectrum from that of each time point of the kinetics.

Infrared spectra were measured during the aggregation process under the same conditions of the above experiments (Figure S3). The development of a prominent band at 1622 cm^−1^ indicative of the formation of β-sheet structure occurs in a rapid phase within 100–120 min (Figure 4). Further development of β-sheet structure occurs in a slower process as a result of fibril formation.

The rapid conformational change to achieve beta-sheet structure was also observed by far-UV CD in a previous work although these data were presented in a normalized form (mean-residue molar ellipticity, see Figure 2b in ref. [23]). The negative CD signal at 215 nm increases very rapidly in a fast phase within about 100 min, indicating a fast conformational change with β-sheet formation even at the lowest protein concentration analyzed (0.36 mM). The initial rates of CD signal development scale approximately with the 1.5 power of the initial protein concentration, suggesting that the appearance of some β-sheet structure occurs prior to the fibril nucleation and is likely to be concomitant to pre-oligomerization. These results clearly indicate the occurrence of a rapid conformational phase previous to nucleation of the amyloid fibrils, which involves partial unfolding of the protein, formation of β-sheet structure and oligomerization.

### Detection of partially-folded species

**Figure 5:**
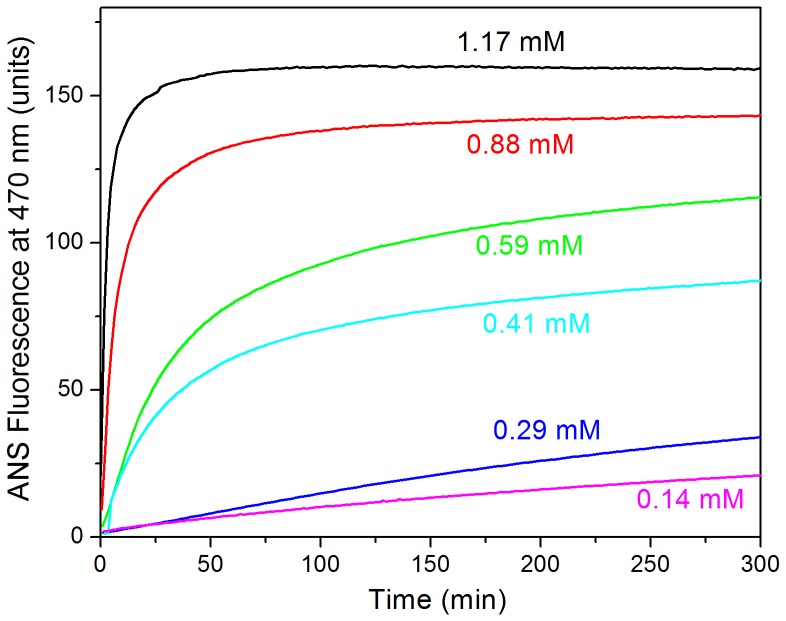
Exposure of hydrophobic surface during the course of aggregation of N47A Spc-SH3. Aggregation was monitored by ANS fluorescence at several initial protein concentrations are indicated along each curve. The concentration of ANS in these experiments was 50 µM.

The formation of oligomers of partially unfolded protein may result in the exposure of hydrophobic surface, which could be monitored using ANS fluorescence. We first followed the process for a protein concentration of 1.26 mM using different ANS concentrations ranging between 10 µM and 400 µM (not shown). The ANS fluorescence intensity develops rapidly in all experiments reaching a plateau. The final ANS fluorescence intensity is approximately proportional to the ANS concentration up to about 100 µM and then tends to saturate. Above 50 µM ANS we found a significant effect of ANS on the rate of aggregation (results not shown). Using 25 µM ANS and varying the protein concentration, we observed considerable dependence of the growth rate of fluorescence intensity and absence of lag phase (Figure 5). The growth of ANS fluorescence intensity occurs earlier than that of ThT fluorescence, indicating rapid exposure of hydrophobic patches preceding nucleation of amyloid structure. Initial rate analysis indicates an apparent order of 3.1 ± 0.5, which is intermediate between the order observed for fibrillation and that of the initial conformational change monitored by far-UV CD. These results suggest that the development of the ANS fluorescence signal is not specific of the appearance of oligomers but it may also report on the formation of amyloid structures. This is supported by separate experiments, in which we found that ANS co-precipitates with the amyloid fibrils indicating strong ANS binding to amyloid structures (results not shown).

### Direct detection of oligomeric species

**Figure 6:**
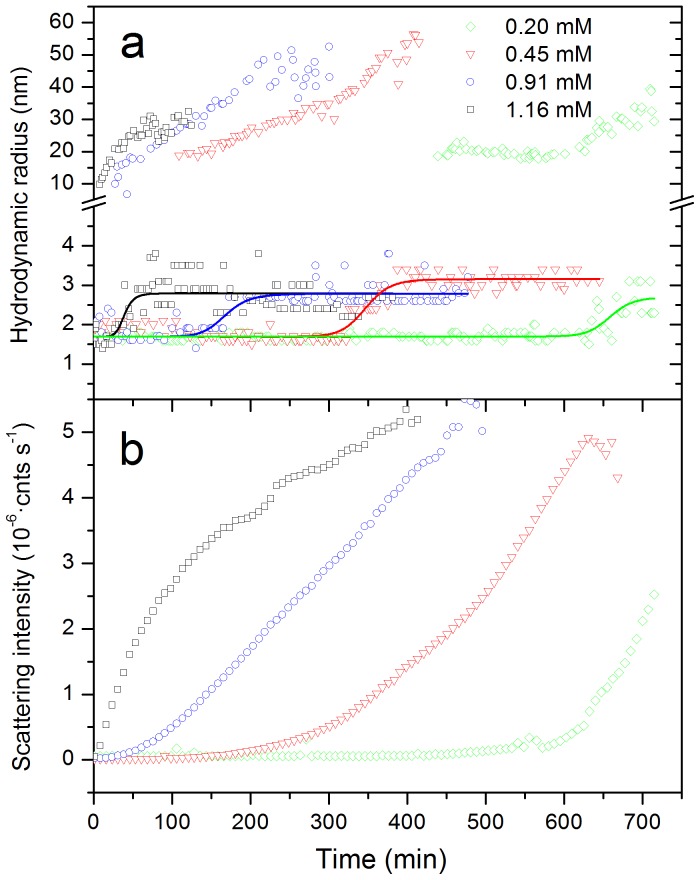
Time course of aggregation of N47A Spc-SH3 monitored by DLS. Several initial protein concentrations were analyzed as indicated. A: Hydrodynamic radii of the two smallest particles visible in the size distributions as a function of the time of incubation. The sigmoidal lines are used only for clarity purposes. B: Time dependence of the scattering intensity.

To investigate the growth rate of aggregates, DLS measurements were performed during the course of the aggregation at several protein concentrations. Figure 6 shows the time evolution of the scattering intensity and the apparent hydrodynamic radii (R_h_) of the smallest particles visible in the size distributions. The changes in the particle size distributions clearly indicate a progressive conversion of the native protein (detected at the early times as a peak with apparent radius of about 1.7 nm) into larger particles (Figure 6A). Growing particles are observed with apparent radii starting from about 10–20 nm and increasing up to 30–50 nm, depending on the initial protein concentration, similarly to that observed for Abeta fibrillation by DLS [28]. In addition, the peak corresponding to the native R_h_ shifts up to ca. 2.7 to 3.0 nm, which suggests the association of a few protein molecules into small oligomers. This shift is considerably delayed as the protein concentration is reduced indicating a slower accumulation of oligomers. The delay of oligomerization is coincident with a similar delay in the growth of scattering intensity (Figure 6B). The lag in the development of scattering intensity is not observed in the kinetics monitored by ThT fluorescence at similar concentrations, which indicates that only small-sized amyloid particles, likely amyloid nuclei, form at early times in the kinetics. The fact that the onset of growth in scattering intensity is concurrent with the oligomerization is suggestive of a condensation of nuclei as the process dominating the growth of large amyloid aggregates.

**Figure 7:**
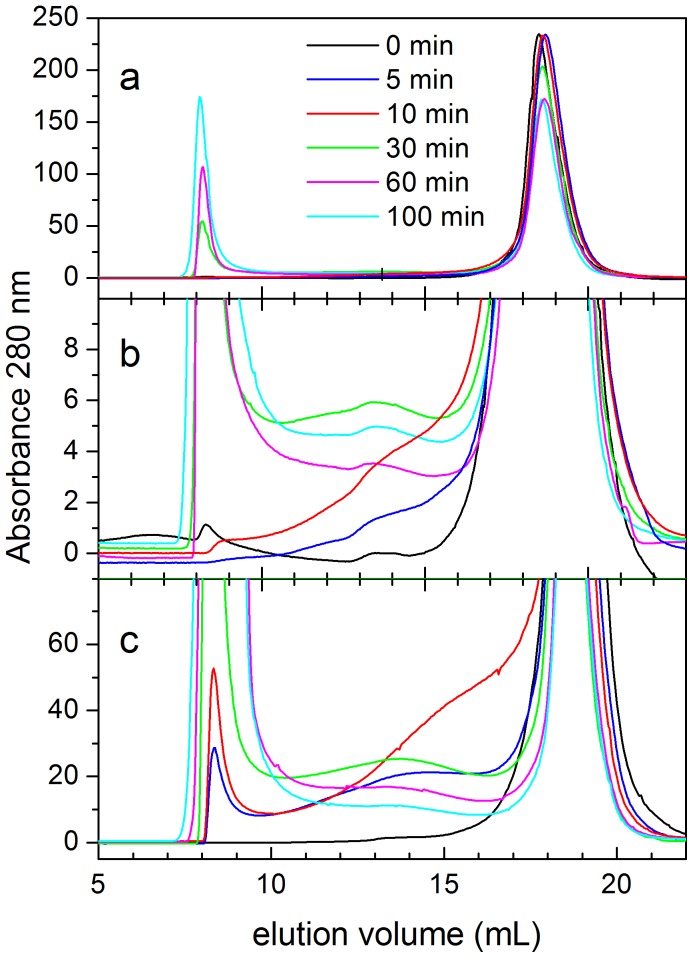
Size-exclusion chromatography analysis of N47A Spc-SH3 during the course of aggregation. Samples were incubated at 37°C during the times indicated along each elution profile prior to the analysis. A: Initial protein concentration of 1.1 mM; B: expansion of the ordinate axis of panel A; C: initial protein concentration of 2.2 mM.

SEC experiments were also made to analyze the mixtures during the early stages of the fibrillation (Figure 7). Samples of 8 mg mL^−1^ (1.1 mM) of protein were incubated at 37°C for several time periods and analyzed in a Superdex S75 column previously equilibrated in the same aggregation buffer and also thermostatized at 37°C (Figure 7A). The elution profile of a non-incubated sample shows a single peak eluting at 18.5 mL, corresponding to the native monomer. After 30 min of incubation, high-molecular weight particles are already formed eluting at the exclusion volume of the column. The amount of these aggregates increases at longer times of incubation while the amount of monomer decreases concomitantly. These species correspond very likely to stable protofibrils or fibrils. In contrast, for protein samples incubated for only 5 min or 10 min there is still no evidence of formation of the high-molecular weight aggregates and most of the protein eluted as monomer. There is however a non-negligible UV absorption in the elution profiles for elution volumes corresponding to intermediate sized particles, suggesting a low population of oligomeric species (see Figure 7B). These low-molecular-weight oligomers cannot be detected at any elution volume if the SEC analysis of the same samples is made at 4°C or 25°C (not shown), indicating reversibility of the oligomerization at low temperature. When we repeated the same experiment at 37°C and double protein concentration (2.2 mM), the presence of small oligomers is much more evident in the profiles and the formation of high-molecular weight particles is faster (Figure 7C). There is no clear peak corresponding to any particular oligomer size but a broad UV absorption between the monomer and the large aggregate peaks. This suggests the existence of a distribution of interconverting oligomers, very likely in equilibrium with the monomers. Since SEC is a separative technique, the dilution that occurs during the chromatography separation would tend to shift the equilibrium towards the monomer.

From these results, we can conclude that early oligomers form rapidly during the kinetics leading to the early accumulation of small amyloid nuclei. The rate of this process is strongly dependent of the protein concentration and shows apparent high-order kinetics.

### Influence of the salt concentration on the nucleation kinetics

Previously, we have reported that the concentration of NaCl strongly affects the nucleation of the N47A Spc-SH3 fibrillation [23]. Here we re-investigated the effect of the salt concentration on the nucleation kinetics by measuring the initial rates of fibrillation at 37°C by ThT fluorescence. Initial rates were measured under each condition only at protein concentrations producing an absence of significant lag phase, indicating a fast amyloid nucleation. As observed before, an increase in NaCl concentration accelerates the nucleation but, interestingly, reduces the apparent order of the kinetics from 6.4 ± 0.3 at 50 mM NaCl to 3.2 ± 0.2 at 200 mM NaCl (Figure 1B, Table 1). This might be in principle interpreted as a reduction in the average size of the critical oligomers preceding the nucleation produced by the increase in the concentration of salt ions. This would be contrary however to the increase in size of oligomers produced by a rise in the concentration of NaCl as observed by DLS in our previous work [23].

### A simple nucleation model to interpret initial aggregation rates

**Figure 8:**
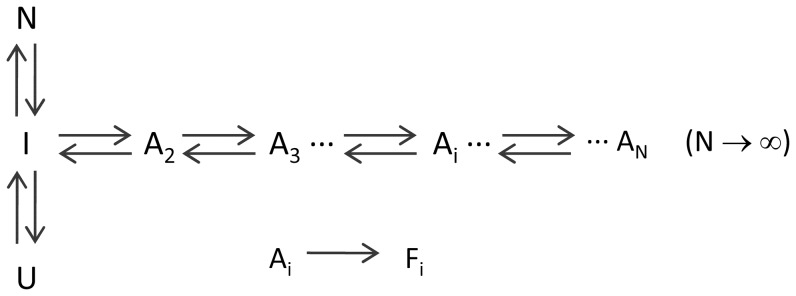
Kinetic scheme for amyloid nucleation. N, I and U represent respectively the native, intermediate and unfolded monomeric states. A_i_ states represent oligomers of i size, where i can take any value from two to infinite. F_i_ represent oligomeric amyloid nuclei. Double arrows indicate rapidly-equilibrated reversible processes and the single arrow indicates a rate-limiting irreversible process.

To understand the observed kinetic effects in terms of a plausible mechanism, we proposed a simple model for amyloid fibril nucleation. This model is based upon previous polymerization models reported in the literature [3], [5], [6], in which partial unfolding and/or linear polymerization processes previous to the rate-limiting steps of aggregation were assumed to be pre-equilibrated, i.e., they can be considered sufficiently fast to be under equilibrium during the process. The model is summarized in Figure 8 and mathematically developed in detail in the Supporting Information (Text S1):

In this model, N and U are respectively the native and the unfolded states of the protein. The state I is a monomeric intermediate prone to intermolecular association in equilibrium with the folded and unfolded states. The species A_i_ are oligomeric aggregates formed by reversible association of intermediate molecules. There is no limit imposed in our model to the maximum size of these oligomers, which will be only determined by the concentration of the associating species and the equilibrium association constants. The nucleation step was modeled as an irreversible conformational change of the protein when it is part of an oligomer, giving rise to an oligomeric amyloid nucleus F_i_ of the same size [6]. We did not impose any restriction to the size of the oligomers that may undergo the nucleation change, except for the fact that nucleation can only occur from oligomeric states. This is different to most aggregation models, which usually assume a unique nucleus size. Since we are specifically modeling the initial rates of the amyloid nucleation process, we assumed that further elongation steps are not significant during the very early stages of the kinetics and do not contribute significantly to the accumulation of amyloid structures. This assumption is reasonable since we are extrapolating the aggregation rates to time zero, where no amyloid nuclei are still formed. In addition, as discussed in the Supporting Information (Text S3), a significant contribution of monomer addition to any nuclei or fibrils formed during the early times would result in upwards curvature of the kinetics, which is not observed under our experimental conditions, except for very slow nucleation conditions, such as at low protein and salt concentration (results not shown).

The equilibrium constants governing the conformational equilibrium of the monomeric protein are defined as:
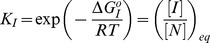
(4a)

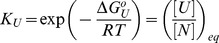
(4b)


And the equilibrium constant for each oligomerization step:
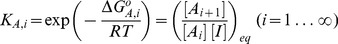
(4c)


We assumed for simplicity that the equilibrium constants of all oligomerization steps are equal (*K_A,i_ = K_A_*) [3]. Using this assumption, it is easy to obtain relatively simple equations relating the total protein concentration, C_0_, to the equilibrium constants and the population of every equilibrium state (see Supplementary Material). For instance, the fraction of protein in the native state, x_N_, can be obtained solving the following equation:
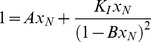
(5)


Where *A = 1+K_U_*, and *B = K_I_·K_A_·C_0_*. Similarly, the pre-equilibrated concentration of monomer in oligomeric species prior to the onset of the aggregation process is given by:
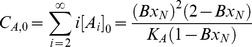
(6)


And the initial rate of conversion of protein to the amyloid state is given by:
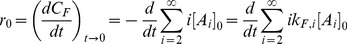
(7)


Here *C_F_* stands for the concentration of protein monomers in amyloid states. *k_F,i_* is the first-order rate constant for the conformational change of an i-sized oligomer to form an amyloid nucleus of equal size. Also for the sake of simplicity, we assumed that the rate constants are approximately independent of the size of the oligomers, i.e. *k_F,i_ ≈ k_F_*. Using this assumption eq. 7 becomes:
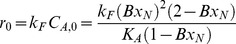
(8)


According to this equation, the initial slopes of the kinetics of amyloid growth –for instance, those measured by ThT fluorescence– will be a function of the first-order rate constant of conformational conversion, the initial protein concentration and the thermodynamic parameters governing the conformational-association equilibrium preceding the rate-limiting nucleation step.

Using equation 8 we simulated the initial rates of amyloid nucleation for an extensive set of values of the model parameters. A summary of the results of these simulations is shown in the Supporting Information (Text S2). First, we simulated the case where the amyloidogenic intermediate is the most stable state under the aggregating conditions (*K_I_*>>1). In this case there are two limiting nucleation regimes: A) Low protein concentrations: C_0_<<K_A_^−1^. Under these conditions the slope of the plot of log r_0_ vs log C_0_ is linear with a slope of 2. This means that most of the protein is in the monomeric state and the nucleation takes place via the dimer, with apparent 2^nd^ order (I ↔ A_2_ → F_2_). B) High protein concentration: C_0_>>K_A_^−1^. In this case, the slope of the double logarithmic plot is 1 because most of the protein pre-equilibrates as oligomers and the process simplifies to a first-order reaction (A_i_ → F_i_). The change from second to first order occurs progressively when C_0_ approaches to K_A_^−1^.

In the case of a stable folded protein, with a low-populated amyloidogenic intermediate, there are two similar limiting regimes: A) Second order for C_0_<<(K_A_·K_I_)^−1^ and B) first order for C_0_>>(K_A_·K_I_)^−1^. For protein concentrations around a critical concentration, C_0_≈(K_A_·K_I_)^−1^, the plot of log r_0_ vs log C_0_ undergoes an inflexion with a transient increase in slope. Interestingly, for the same critical concentration, the maximum slope depends on the relative values of K_I_ and K_A_, i.e., on the stability of the monomeric intermediate compared to that of the oligomers. Low values of K_I_ and high association constant, K_A_, give rise to a steep inflexion with high apparent reaction orders. This is due to a quite pronounced shift in the oligomerization pre-equilibrium as the protein concentration rises.

Changes in the values of the rate constant of the conformational change, k_F_, result only in a vertical displacement of the double logarithmic plot. The effect of changes in the global unfolding equilibrium constant, K_U_, is relatively small because it affects only marginally the relative population of I and the corresponding oligomerization equilibrium.

Using the equations of this model, we have fitted the experimental initial rates of amyloid nucleation measured by ThT fluorescence as a function of the protein concentration. In this analysis we assumed that only the amyloid nuclei are detected by thioflavin T fluorescence. An important problem arising when fitting these data is that the theoretical equations depend on four independent parameters (K_A_, K_I_, K_U_ and k_F_). To reduce their number, we fixed the values of K_U_ using the experimental value at 37°C derived from the analysis of the thermal unfolding by DSC at low protein concentration [23]. Another difficulty in this analysis is that the interval of protein concentrations and the range of experimentally accessible initial rates are limited. For instance, at 50 mM NaCl, the fibril nucleation is fast only at high protein concentrations, whereas below 1 mM the aggregation kinetics shows a considerable lag phase [23]. The range of experimental data is therefore quite limited for this condition.

Individual fits at the three different salt concentrations describe very well the data but the dependency between the fitting parameters is high. We found that the fits converge to similar values for K_A_ for the different NaCl concentrations and the three sets of initial rates could be globally fitted using a common value for this parameter (Figure 1b), which suggests that the association equilibrium constant does not change much with the concentration of NaCl. The parameters resulting from this analysis are shown in Table 2. The increase in salt concentration stabilizes the amyloidogenic intermediate relative to the native and unfolded states, thus increasing its population. The rate constant of the conformational conversion of oligomers into amyloid nuclei is also considerably increased with salt concentration.

**Table 2:** Fitting parameters derived from the analysis of initial nucleation rates at 37°C.

[NaCl]	K_U_ a	K_A_ (×10^−3^)	K_I_	k_F_ (min^−1^×10^−3^)
N47A Spc-SH3				
50 mM	0.071 (fixed)	246 ± 50	0.0024 ± 0.0010	2.0 ± 3.6
100 mM	0.117 (fixed)		0.0058 ± 0.0010	17.6 ± 2.8
200 mM	0.165 (fixed)		0.0109 ± 0.0018	38.7 ± 7.3
N47A-K43A Spc-SH3				
100 mM	0.495 (fixed)	246 (fixed)	0.036 ± 0.010	34.6 ± 7.8

a Calculated from the unfolding curves measured by DSC. Data of N47A Spc-SH3 were taken from reference [23].

To further validate our nucleation model, we studied the aggregation kinetics of a double mutant of the Spc-SH3 domain (N47A+K43A). The second mutation K43A destabilizes strongly the native state. The far-UV CD spectrum indicates a partially unfolded conformation (Figure S5a) and the DSC thermogram obtained at a low protein concentration shows a weak and broad unfolding transition (Figure S5b), consistent with a low structural cooperativity. This double mutant forms fibrils much faster than the single mutant N47A in the presence of 100 mM NaCl as observed by TEM (results not shown). Initial rates of nucleation were measured by ThT fluorescence (Figure 1b). Under these conditions, there is a rapid growth of ThT fluorescence indicating a fast formation of amyloid nuclei. The plot of log r_0_ vs log C_0_ has a slope close to one over most of the concentration range explored. This is consistent with a kinetic regime where the amyloid nucleation takes place from the oligomers, i.e., C_0_>>(K_A_·K_I_)^−1^. In fact, DLS indicates that this double mutant domain forms oligomers even at 15°C and at relatively low concentrations (Figure S5c). SEC analysis at 37°C also shows a considerable population of oligomers prior to the incubation and during the very early times of the aggregation kinetics (Figure S5d). Fitting the initial aggregation rates using our model yielded a considerably higher K_I_ for this mutant, mainly due to a strong destabilization of the native state. We needed however to fix the K_A_ value in this fitting because the data did not contain enough information for an accurate determination of all the fitting parameters (Table 2). Nevertheless, these results strongly support the validity of our model and its power to provide thermodynamic and kinetic data characterizing the amyloid nucleation process.

## Discussion

Here we have shown that amyloid fibrillation of the N47A Spc-SH3 mutant domain takes place following apparent high-order irreversible kinetics, as a result of oligomeric precursors preceding the fibril nucleation event. This high-order kinetics is confirmed by fitting of aggregation kinetics followed by 2D-NMR or near-UV CD, which selectively monitor the concentration of native monomers. On the other hand, the kinetics followed by far-UV CD and ANS fluorescence show that partial unfolding of the native protein involving secondary structure changes and the exposure of hydrophobic patches takes place significantly faster than nucleation and with lower apparent order. This implies that these early conformational changes are, at least in part, associated to the formation of the pool of oligomeric precursors themselves. These oligomers are likely to be low-populated, unstable and interconverting species. Formation of transient oligomeric intermediates has been previously reported as a crucial event preceding amyloid fibrillation in many proteins. For instance, oligomeric intermediates formed at low pH above a critical concentration of amyloid-beta 1–40 were proposed to have micellar properties and be in equilibrium with the monomeric protein [28]. Using photo-induced cross linking it was observed that the Abeta 1–40 peptide undergoes a rapid oligomerization equilibrium involving monomer, dimer, trimer, and tetramer during the pre-nucleation phase of fibril assembly [8]. Insulin fibrillation takes also place by rapid formation of soluble oligomers followed by a slower transition into a second distinct class of oligomers that lead to amyloid fibrils [29]. Single-molecule fluorescence methods have also shown a rapid and transient increase in the population of oligomeric species during the early stages of PI3–SH3 aggregation [7]. These early oligomers formed a highly heterogeneous size distribution. All these studies and many others show common features for early stages of amyloid formation: i) early oligomers are structurally unstable and interconverting species with heterogeneous sizes; ii) the oligomerization pre-equilibrates rapidly from the monomers prior to the nucleation event; iii) the oligomers then convert into amyloid structures, which is the rate limiting step in fibrillation. These general characteristics are in good agreement with our results and constitute the basis of the presented model of amyloid nucleation.

To monitor the initial aggregation rates we selected deliberately the conditions under which the conversion of native N47A Spc-SH3 into fibrils takes place without a lag phase due to a rapid formation of amyloid nuclei. The advantage of this approach is that the earliest times of the kinetic traces followed by ThT fluorescence are mainly sensitive to the nucleation event. Similar lag-free aggregation kinetics was previously observed by Dobson and coworkers [11] for the PI3-SH3 domain at pH 3.6, whereas fibrillation showing a lag phase occurs at lower pH. These authors invoked a “nucleated conformational conversion” mechanism to explain their fibrillation kinetics in the absence of lag phase, similar to that proposed here. This mechanism was previously proposed for the prion protein Sup35 [9], which was reported to form structurally fluid oligomeric complexes that appear to be crucial intermediates in the *de novo* amyloid nucleation. Fibrillation of β2-microglobulin into disordered worm-like fibrils or short rod-like fibrils has also been shown to occur without a lag-phase by an aggregation pathway, which is different to that of the relatively rigid long-straight fibrils classically associated with amyloid [30]. The partitioning between these different competing pathways is modulated by environmental conditions and occurs during the earliest conformational and association phenomena, which direct subsequent events leading to different types of aggregates.

Since the early work of Oosawa et al. [3] many theoretical models to describe quantitatively the kinetics of protein aggregation have been proposed with the aim to obtain kinetic and thermodynamic parameters characterizing the critical steps of the process (see [5] for a comprehensive review). Models were developed to fit the nucleation-dependent kinetic curves typical of protein aggregation and were usually formulated with the assumption of an aggregation nucleus of a specific size [6], [31]–[33]. Recently, Roberts and coworkers have proposed a combined Lumry-Eyring and Nucleated Polymerization (LENP) model [6], [34], in which the aggregation rates are considered slow compared to the folding-unfolding equilibrium. They model the nucleation event as a conformational rearrangement of x-sized oligomers into x-sized nuclei. Interestingly, by exploring different qualitative kinetic regimes, they showed that their model is a generalization of many previously proposed nucleation and growth models for aggregation. One of their main findings is that under the scenarios where nucleation is fast, the apparent kinetics of monomer depletion follows x-order kinetics. Our finding of high order kinetics is in agreement with the predictions of the LENP model, which supports the validity of our assumption of fast nucleation. Apparent sizes of the dominant amyloid nuclei ranging between 3 and 6 molecules would be derived from the apparent order of the kinetics depending on the conditions.

In spite of this agreement, our nucleation model does not imply any assumption of a specific size for the aggregation nuclei. In fact, we model the nucleation event as a conformational change taking place within any preformed oligomer irrespective of their size. The size distribution of the oligomers is however controlled by the thermodynamic parameters of the process, i.e., the stability of the amyloidogenic intermediate, the equilibrium constant for the oligomerization and the protein concentration.

In a recent work, Vitalis and Pappu [35] have reanalyzed the aggregation mechanism of polyglutamine peptides using a model that also accounts for the possibility of nucleation from a pool of heterogeneous oligomers, similarly to the model proposed here. They clarified previous observations of fractional and/or negative values of estimates of the critical nucleus size arising from the assumption of a homogeneous nucleation model. The model proposed by these authors for early aggregation shares with the nucleation model proposed here two major assumptions: First, a rapidly pre-equilibrated distribution of oligomers is formed by successive monomer addition; second, the nucleation event occurs by a rate-limiting conformational conversion of oligomers into pre-fibrillar species. On the other hand, there are several mathematical and conceptual differences between the two models: Vitalis and Pappu do not analyze the initial aggregation rates but they adapt their model to the analysis of early aggregation kinetics as originally developed by Ferrone [33], in which they analyze the slope of extent of aggregation plotted versus t^2^. They also take into account the elongation of the fibrillar aggregates via monomer addition, whereas we neglected its contribution in our model as discussed above and in Text S3 of the Supporting Information. In addition, these authors limited in their analysis the maximum oligomer size and the minimum size of oligomers that are competent for conformational conversion. Finally, they do not consider the existence of a folding-unfolding equilibrium for the monomer.

In spite of these differences, some of their fundamental conclusions are similar to ours. They highlight the importance of the factors controlling the oligomer distribution in determining the apparent oligomer size in the analysis of the kinetics is made assuming a single nucleus size. In particular, if the process nucleates under conditions where large fractions of the aggregating material are present as soluble oligomers that are competent to promote fibril formation, then the apparent nucleus size may results in quite anomalous values (fractional or even negative in their analysis).In fact, using the equations of the model and the parameters of Table 2, we could calculate the average size and the size distributions of the pre-equilibrated oligomers for each concentration of protein (see Figure S6). It is evident that the size of the oligomers and their population depend quite dramatically on the total protein concentration. At low concentrations there are mainly dimers at very low populations. As concentration increases, the overall oligomer population increases and the size distributions become shifted toward larger sizes and considerably broadened. An increase in the NaCl concentration strongly enhances oligomerization. Importantly, the experimental order of the kinetics does not need to be correlated with the average oligomer size, except if the measurements are made near the critical concentration. For instance, at 50 mM NaCl concentration, the N47A Spc-SH3 mutant shows an apparent order of 6.4, which is in very good agreement with the average sizes of the oligomers predicted by our model (between 4 and 8 in the protein concentration interval explored) but, despite this coincidence, the size distributions are quite broad at these protein concentrations. In contrast, the kinetics of the K43A-N47A double mutant were studied well above its critical concentration and even though the aggregation nucleates from a wide variety of oligomer sizes the experimental order is close to one. The observation of concentration-independent nucleation at high protein concentration appears to be a general property of polymerization models due to the fact that under this regime the nuclei are no longer the least thermodynamically unstable species, as described by Powers and Powers [36].

The direct detection of the oligomeric precursors of nucleation has proved extremely difficult, due to their instability and the fact that they are not usually the dominant species in the aggregating solution. Here we used DLS and SEC to analyze the mixtures during the course of the aggregation. DLS shows the formation of oligomeric particles with average hydrodynamic radii of about 2.7 to 3.0 nm and SEC is also able to detect a low population of oligomeric species at early aggregation times but only when the samples were analyzed at 37°C and at very high protein concentrations. In contrast, the oligomers are clearly present for the K43A-N47A double mutant even prior to aggregation, as demonstrated by DLS and SEC. It is likely, however, that the oligomer fraction detected during aggregation includes also stable amyloid nuclei, which would not dissociate during the SEC analysis. Nevertheless, the existence of this range of oligomeric species is fully consistent with the observed aggregation kinetics.

**Figure 9:**
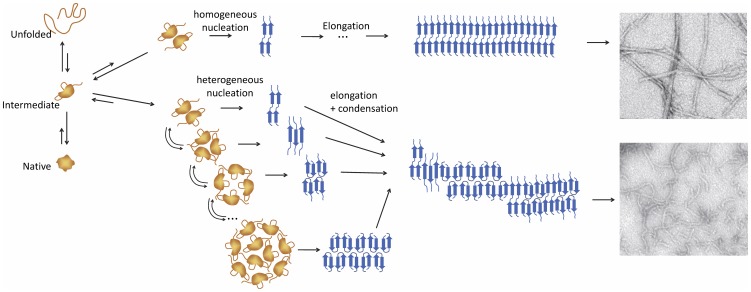
Simplified model of amyloid fibril nucleation. Schematic illustration based on the results of this work of how the environmental conditions could modulate the mechanism amyloid nucleation. Non-amyloid partially-unfolded molecules and oligomers are represented in orange, whereas amyloid nuclei and fibrils are represented in blue. Top and bottom electron micrographs correspond respectively to twisted-straight amyloid fibrils and curly fibrils of N47A Spc-SH3 obtained under different conditions of nucleation.

The possibility of nucleation from oligomers of different sizes, which are modulated by the experimental conditions, could have profound implications in understanding the mechanism of amyloid aggregation. This is schematically illustrated in Figure 9. The conformational conversion of the polypeptide chain into amyloid structure may be constrained differently within oligomers of different sizes, giving rise to a structural variability in the nuclei and a consequent polymorphism of the final amyloid structures modulated by the conditions [23], [30], [37]. Also, the amount and size distribution of nucleating oligomers would affect strongly all subsequent fibrillation stages. Conditions disfavoring oligomerization, such as low salt or low protein concentration, would promote a slower nucleation via smaller oligomers. As a consequence, less abundant and more homogeneous nuclei would preferentially grow via monomer addition into more ordered filaments that could then assemble into mature twisted fibrils. On the other hand, rapid accumulation of a heterogeneous distribution of amyloid nuclei could promote aggregation via condensation or growth of heterogeneous fibrils via monomer addition to a diversity of structural templates, in any of these cases giving rise to more disordered fibrillar aggregates [11] or even amorphous aggregates as we observe for N47A Spc-SH3 in the presence of 300 mM NaCl (results not shown).

An important advantage of our kinetic approach is its mathematical and experimental simplicity. The measurement of initial aggregation rates are relatively straightforward and do not need the recording of long aggregation kinetics, allowing the exploration of a larger number of experimental variables. Also, the equations describing the initial rates are very simple and complicated numerical integration procedures are not necessary to fit the data. This method could be applicable to any protein aggregation process as long as the kinetics does not show a lag phase and the initial rates are representative of the formation of the earliest amyloid structures.

Our analysis provides important thermodynamic and kinetic magnitudes allowing us to rationalize the nucleation event. This is of chief importance to understand the factors governing the stability of early amyloidogenic species. The amyloidogenic monomeric intermediate of N47A Spc-SH3 is a very unstable state, likely an ensemble of partially unfolded species. Its stability is much lower even than the fully unfolded state under the aggregation conditions. It has however a very strong propensity to oligomerize, as indicated by the large association constant K_A_. As a result, the concentration of I is very low under all conditions, but the factors affecting its stability are key to modulate the amount and distribution of oligomers that are competent to nucleate aggregation. This is crucial to understand the mechanisms by which proteins maintain solubility and avoid deleterious aggregation processes. Protein sequences have evolved to maintain a high structural cooperativity of the native states, favoring either the native or the globally unfolded state under all conditions and reducing the population of aggregation-prone partially unfolded species. A reduction in the global unfolding cooperativity produced by mutations has been shown to enhance amyloidogenicity [38].

Our kinetic approach could be also relevant for the case of intrinsically disordered proteins, such as Abeta or α-synuclein, because their most populated soluble states are not necessarily the amyloidogenic ones. It has been shown that disordered polypeptide chains must partially refold to initiate amyloid aggregation [39] achieving a high-energy, partially-folded conformation, which then is able to oligomerize and aggregate. This is similar to what we observed for the double mutant K43A-N47A Spc-SH3. The soluble monomeric state is probably an ensemble of conformations with low unfolding cooperativity but the amyloidogenic conformation does not need to be the most populated one but just a subfraction of this ensemble.

Another important result is that an increase in the NaCl concentration appears to produce a selective stabilization of the monomeric intermediate, I, and not an enhancement of its association tendency. NaCl also increases the rate constant of the conformational conversion to amyloid structure. Salt ions may affect protein aggregation by a number of mechanisms but how each specific molecular stage is influenced by ions is still not known. Simple Debye-Hückel screening of electrostatic interactions has been ruled out as a main contributor to aggregation for several systems [40], [41], although it plays a role on fibrillation of α-synuclein at low ionic strengths [42]. Preferential anion binding to the protein groups has been proposed as a main mechanism promoting fibrillation of β2-microglobulin at acid pH, because the aggregation enhancement produced by different ions is ordered according to the electroselectivity series [43]. Similar observations were made for the fibrillation of glucagon [40] and mouse prion protein [44] both at acid pH. In contrast, the effect of ions on α-synuclein fibrillation has been found to follow the Hofmeister series, suggesting an important role of hydration effects [42]. The aggregation of Abeta1–40 at pH 9 seems to depend on a combination of both effects [41]. The result of our analysis ascribes the effect of salt ions mainly to a relative stabilization of the amyloidogenic monomer, which is partially unfolded and exposes a significant amount of hydrophobic surface. Also, at the mildly acid pH of our experiments the protein is positively charged. At the relatively low NaCl concentrations used in these experiments and given that the Na^+^ and Cl^−^ ions are neither strong “salting-out” or “salting-in” ions [45], it is unlikely that the intermediate is primarily stabilized by a Hofmeister effect. In fact, the fully unfolded state exposing more surface area is less stabilized by NaCl than the intermediate. Preferential binding of chloride anions to the intermediate relative to the native state could be playing a role in compensating the net positive charge, reducing electrostatic repulsion and thus favoring oligomerization. A more profound investigation of the effect of different salt ions is however necessary to clarify these effects.

## Conclusions

We have shown that the amyloid nucleation kinetics of the Spc-SH3 domain obeys to a high-order irreversible kinetics. The analysis of the aggregation kinetics by a variety of biophysical methods has allowed us to infer that the earliest stages of the amyloid nucleation process imply a pre-equilibrium involving oligomerization of a partially-unfolded intermediate followed by a conformational conversion of the polypeptide chain within the oligomers. The latter process leads to formation of the amyloid nuclei. A simple mathematical model can account very well for the experimental initial rates of aggregation and their dependence with the concentration of protein. The model has allowed us to derive thermodynamic magnitudes characterizing the highly unstable amyloidogenic intermediate and its oligomers. It is shown that environmental conditions such as protein or salt concentration can strongly affect the stability and population of the precursors, leading to different nucleation scenarios that may in turn result in diverse aggregation pathways and fibril morphologies. The kinetic approach presented here may be applicable to characterize the amyloidogenic intermediates and oligomeric precursors of amyloid aggregation in disease-related proteins.

## Supporting Information

Figure S1 **Linearity of the thioflavine T (ThT) fluorescence as a function of the concentration of amyloid fibrils.** Amyloid fibrils of N47A mutant of the Spc-SH3 were prepared by in incubation at 37°C in 100 mM glycine buffer, 100 mM NaCl pH 3.2, isolated by centrifugation and resuspended in the same buffer for ThT assay.
(PDF)

Figure S2 **Near-UV CD spectra of N47A Spc-SH3 in the native state and the amyloid fibrillar state.** Spectra were recorded at the same protein concentration at 25°C in 100 mM glycine buffer, 100 mM NaCl pH 3.2.
(PDF)

Figure S3 **Infrared spectra of N47A Spc-SH3 recorded during the aggregation at 37°C.** Buffer was 100 mM glycine buffer, 100 mM NaCl pH 3.2 and the protein concentration was 1.59 mM.
(PDF)

Figure S4 **Overlay of ^15^N-^1^H HSQC NMR spectra of N47A Spc-SH3 at different times of aggregation at 37°C.** Buffer conditions were the same as in Figure S3, except for the use of per-deuterated glycine and the addition of 10% D_2_O for field lock. The concentration of ^15^N-labelled protein was 1.1 mM (8 mg mL^−1^). The three spectra have been plotted using a single contour level and the same intensity threshold. The times of incubation are: 3 min (blue); 50 min (green) and 1957 min (red). The numbers next to each cross-peak indicate the residue number assignment.
(PDF)

Figure S5 **Structure, stability and oligomerization state of the K43A-N47A Spc-SH3 double mutant.** a) Far-UV CD spectrum in comparison with the N47A single mutant; b) thermal unfolding monitored by DSC; c) Apparent hydrodynamic radius determined at 15°C at different concentrations as indicated. The vertical dashed line indicates the radius of the native Spc-SH3 domain; d) Size-exclusion chromatography at 37°C at two different concentrations as indicated. The peak eluting at ca. 20 mL corresponds to the monomer and the excluded volume is about 8.5 mL.
(PDF)

Figure S6 **Simulations of oligomer size distributions.** Average size distributions were calculated using the parameters derived from the fitting of the experimental initial rates using the equations of the model (see main manuscript text). (a) Average size of the oligomers as a function of the total protein concentration; (b) Average size of all the particles present in solution; (c) Size distribution of the oligomers at 1 mM of total protein concentration; (d) Size distribution of the oligomers at the critical concentration, (K_A_·K_I_)^−1^, obtained under each experimental condition.
(PDF)

Text S1 **Model of amyloid nucleation preceded by partial unfolding and oligomerization pre-equilibrium.**
(PDF)

Text S2 **Model simulations.**
(PDF)

Text S3 **Contribution of fibril elongation by monomer addition to the early kinetics.**
(PDF)
