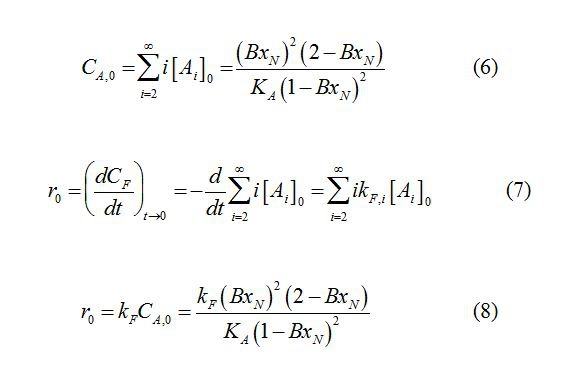# Correction: Characterization of Oligomers of Heterogeneous Size as Precursors of Amyloid Fibril Nucleation of an SH3 Domain: An Experimental Kinetics Study

**DOI:** 10.1371/annotation/dbb84118-9ada-43e4-8734-8f8322be1a59

**Published:** 2014-01-07

**Authors:** David Ruzafa, Bertrand Morel, Lorena Varela, Ana I. Azuaga, Francisco Conejero-Lara

There are errors in Equations 6, 7, and 8 contained in the Results section. Please see the corrected equations here: 

**Figure:**