# Supplementary material for: Characterization of Oligomers of Heterogeneous Size as Precursors of Amyloid Fibril Nucleation of an SH3 Domain: An Experimental Kinetics Study
Source: PLoS One. 2012 Nov 27;7(11):e49690. doi: 10.1371/journal.pone.0049690 (PMC3507826; doi:10.1371/journal.pone.0049690)
Supplement: Figure S1 — Linearity of the thioflavine T (ThT) fluorescence as a function of the concentration of amyloid fibrils. Amyloid fibrils of N47A mutant of the Spc-SH3 were prepared by in incubation at 37°C in 100 mM glycine buffer, 100 mM NaCl pH 3.2, isolated by centrifugation and resuspended in the same buffer for ThT assay. (PDF) [file pone.0049690.s001.pdf]

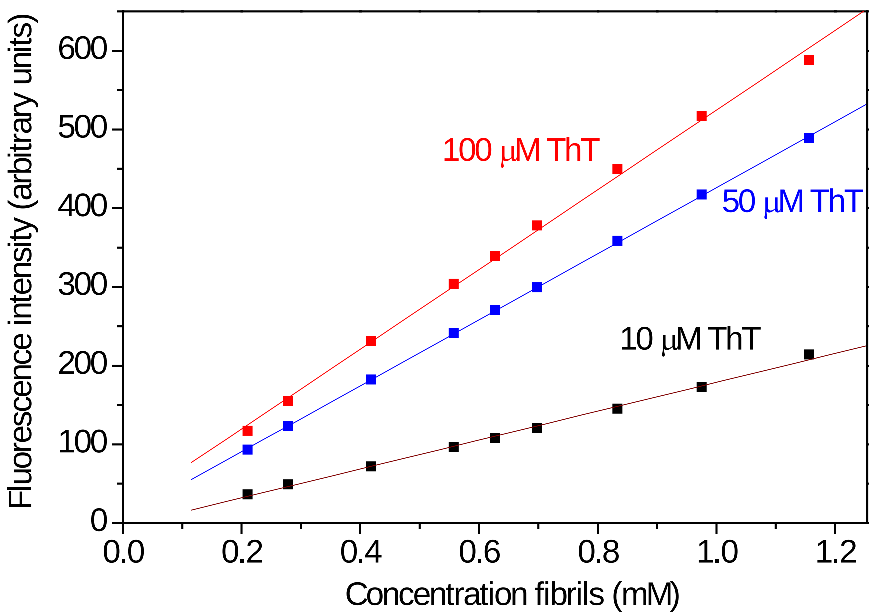

Figure S1. Linearity of the thioflavine T (ThT) fluorescence as a function of the concentration of amyloid fibrils. Amyloid fibrils of N47A mutant of the Spc-SH3 were prepared by in incubation at 37ºC in 100 mM glycine buffer, 100 mM NaCl pH 3.2, isolated by centrifugation and resuspended in the same buffer for ThT assay.
