# Supplementary material for: Characterization of Oligomers of Heterogeneous Size as Precursors of Amyloid Fibril Nucleation of an SH3 Domain: An Experimental Kinetics Study
Source: PLoS One. 2012 Nov 27;7(11):e49690. doi: 10.1371/journal.pone.0049690 (PMC3507826; doi:10.1371/journal.pone.0049690)
Supplement: Figure S2 — Near-UV CD spectra of N47A Spc-SH3 in the native state and the amyloid fibrillar state. Spectra were recorded at the same protein concentration at 25°C in 100 mM glycine buffer, 100 mM NaCl pH 3.2. (PDF) [file pone.0049690.s002.pdf]

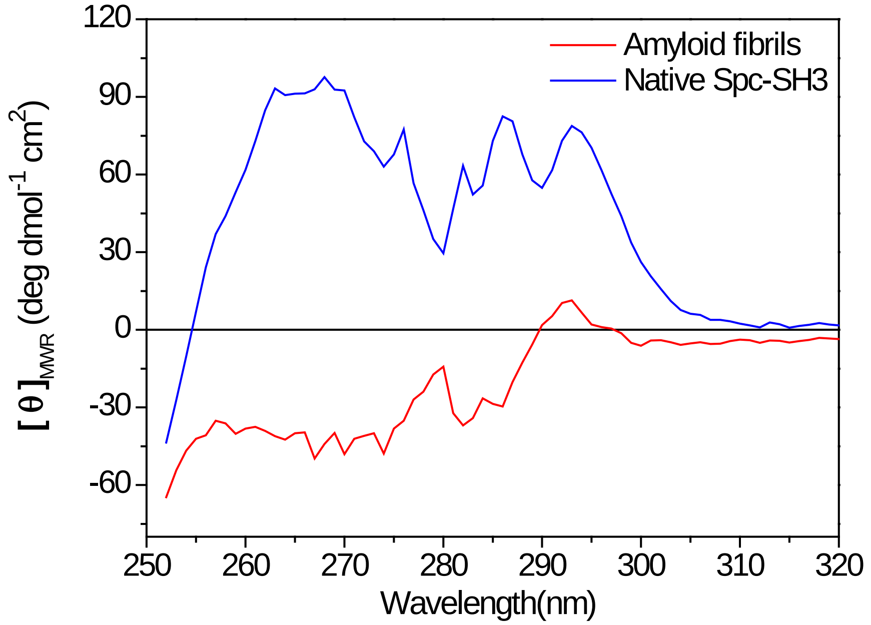

Figure S2: Near-UV CD spectra of N47A Spc-SH3 in the native state and the amyloid fibrillar state. Spectra were recorded at the same protein concentration at 25ºC in 100 mM glycine buffer, 100 mM NaCl pH 3.2.
