# Supplementary material for: Characterization of Oligomers of Heterogeneous Size as Precursors of Amyloid Fibril Nucleation of an SH3 Domain: An Experimental Kinetics Study
Source: PLoS One. 2012 Nov 27;7(11):e49690. doi: 10.1371/journal.pone.0049690 (PMC3507826; doi:10.1371/journal.pone.0049690)
Supplement: Figure S3 — Infrared spectra of N47A Spc-SH3 recorded during the aggregation at 37°C. Buffer was 100 mM glycine buffer, 100 mM NaCl pH 3.2 and the protein concentration was 1.59 mM. (PDF) [file pone.0049690.s003.pdf]

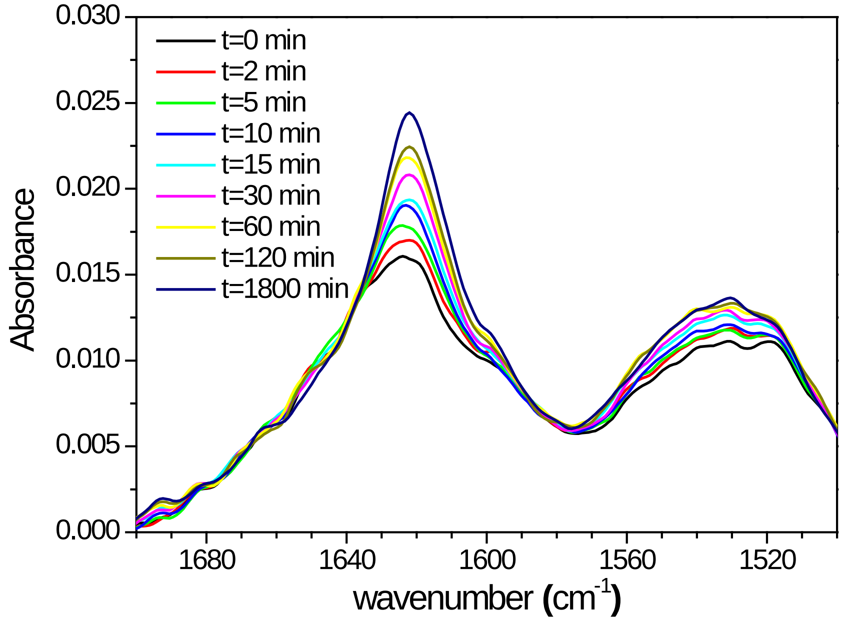

Figure S3: Infrared spectra of N47A Spc-SH3 recorded during the aggregation at 37ºC. Buffer was 100 mM glycine buffer, 100 mM NaCl pH 3.2 and the protein concentration was 1.59 mM.
