# Supplementary material for: Characterization of Oligomers of Heterogeneous Size as Precursors of Amyloid Fibril Nucleation of an SH3 Domain: An Experimental Kinetics Study
Source: PLoS One. 2012 Nov 27;7(11):e49690. doi: 10.1371/journal.pone.0049690 (PMC3507826; doi:10.1371/journal.pone.0049690)
Supplement: Figure S4 — Overlay of 15N-1H HSQC NMR spectra of N47A Spc-SH3 at different times of aggregation at 37°C. Buffer conditions were the same as in Figure S3, except for the use of per-deuterated glycine and the addition of 10% D2O for field lock. The concentration of 15N-labelled protein was 1.1 mM (8 mg mL−1). The three spectra have been plotted using a single contour level and the same intensity threshold. The times of incubation are: 3 min (blue); 50 min (green) and 1957 min (red). The numbers next to each cross-peak indicate the residue number assignment. (PDF) [file pone.0049690.s004.pdf]

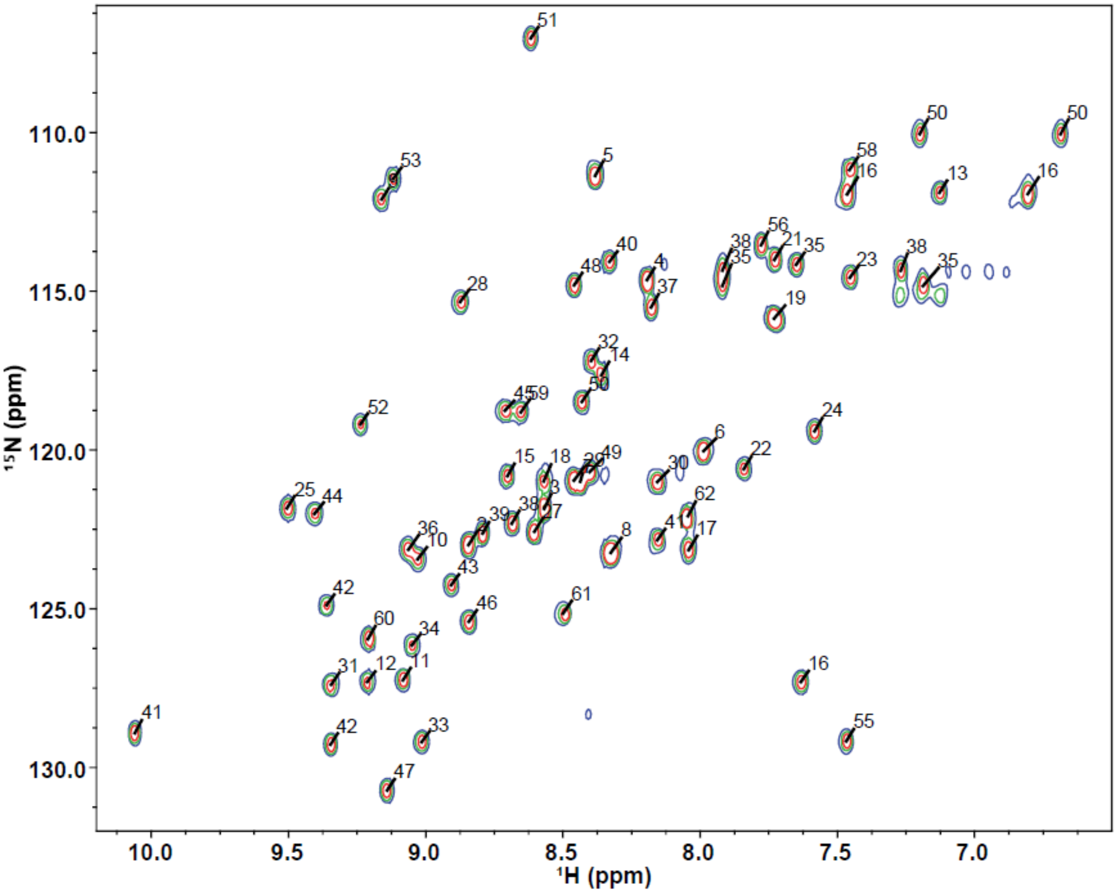

Figure S4: Overlay of ^{15}N-^{1}H HSQC NMR spectra of N47A Spc-SH3 at different times of aggregation at 37ºC. Buffer conditions were the same as in Figure S3, except for the use of per-deuterated glycine and the addition of 10% D_2O for field lock. The concentration of ^{15}N-labelled protein was 1.1 mM (8 mg mL^{-1}). The three spectra have been plotted using a single contour level and the same intensity threshold. The times of incubation are: 3 min (blue); 50 min (green) and 1957 min (red). The numbers next to each cross-peak indicate the residue number assignment.
