# Supplementary material for: Characterization of Oligomers of Heterogeneous Size as Precursors of Amyloid Fibril Nucleation of an SH3 Domain: An Experimental Kinetics Study
Source: PLoS One. 2012 Nov 27;7(11):e49690. doi: 10.1371/journal.pone.0049690 (PMC3507826; doi:10.1371/journal.pone.0049690)
Supplement: Figure S5 — Structure, stability and oligomerization state of the K43A-N47A Spc-SH3 double mutant. a) Far-UV CD spectrum in comparison with the N47A single mutant; b) thermal unfolding monitored by DSC; c) Apparent hydrodynamic radius determined at 15°C at different concentrations as indicated. The vertical dashed line indicates the radius of the native Spc-SH3 domain; d) Size-exclusion chromatography at 37°C at two different concentrations as indicated. The peak eluting at ca. 20 mL corresponds to the monomer and the excluded volume is about 8.5 mL. (PDF) [file pone.0049690.s005.pdf]

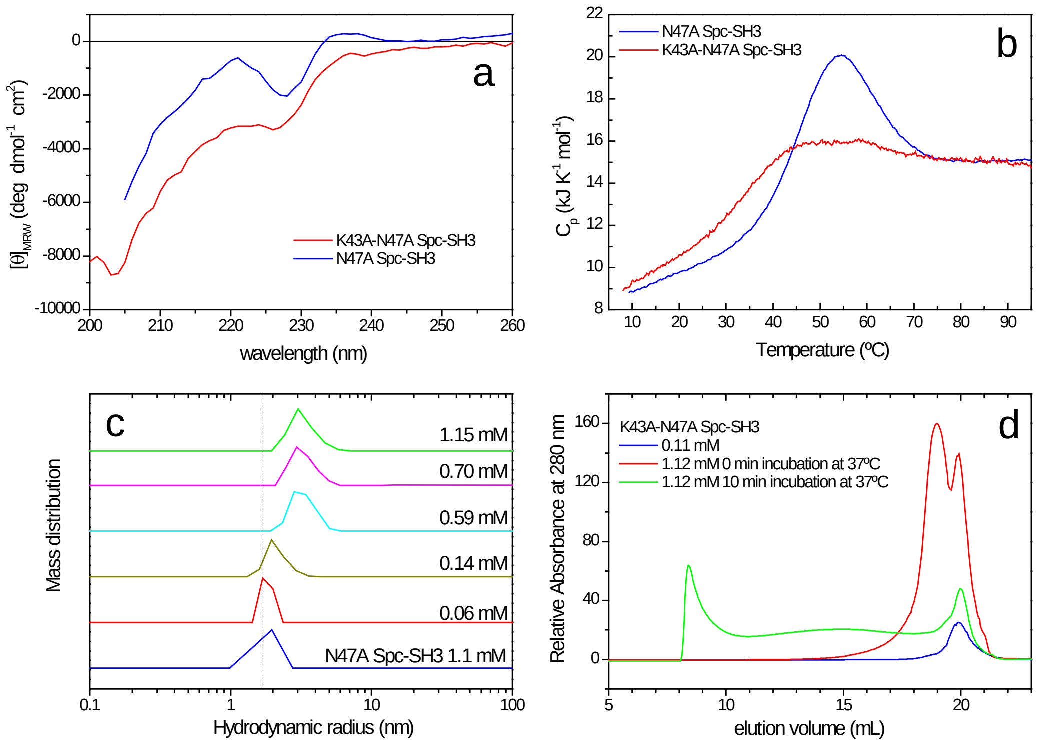

Figure S5: Structure, stability and oligomerization state of the K43A-N47A Spc-SH3 double mutant. a) Far-UV CD spectrum in comparison with the N47A single mutant; b) thermal unfolding monitored by DSC; c) Apparent hydrodynamic radius determined at 15ºC at different concentrations as indicated. The vertical dashed line indicates the radius of the native Spc-SH3 domain; d) Size-exclusion chromatography at 37ºC at two different concentrations as indicated. The peak eluting at ca. 20 mL corresponds to the monomer and the excluded volume is about 8.5 mL.
