# Supplementary material for: Characterization of Oligomers of Heterogeneous Size as Precursors of Amyloid Fibril Nucleation of an SH3 Domain: An Experimental Kinetics Study
Source: PLoS One. 2012 Nov 27;7(11):e49690. doi: 10.1371/journal.pone.0049690 (PMC3507826; doi:10.1371/journal.pone.0049690)
Supplement: Figure S6 — Simulations of oligomer size distributions. Average size distributions were calculated using the parameters derived from the fitting of the experimental initial rates using the equations of the model (see main manuscript text). (a) Average size of the oligomers as a function of the total protein concentration; (b) Average size of all the particles present in solution; (c) Size distribution of the oligomers at 1 mM of total protein concentration; (d) Size distribution of the oligomers at the critical concentration, (KA·KI)−1, obtained under each experimental condition. (PDF) [file pone.0049690.s006.pdf]

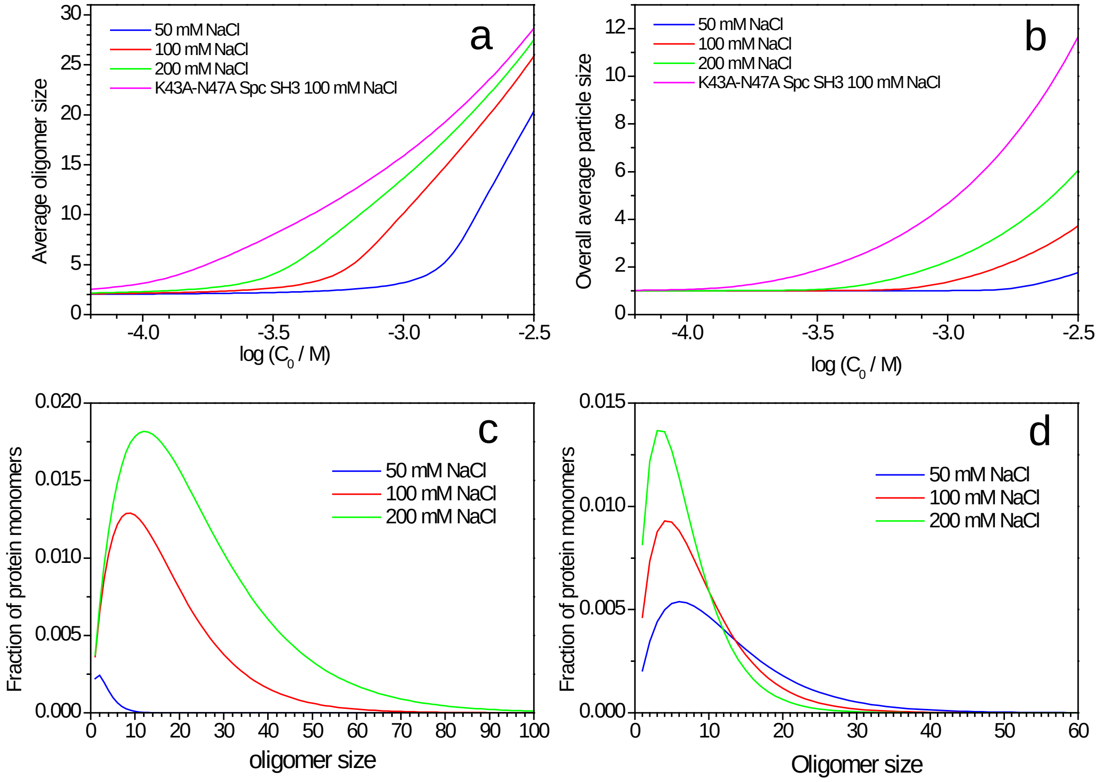

Figure S6: Simulations of oligomer size distributions. Average size distributions were calculated using the parameters derived from the fitting of the experimental initial rates using the equations of the model (see main manuscript text). (a) Average size of the oligomers as a function of the total protein concentration; (b) Average size of all the particles present in solution; (c) Size distribution of the oligomers at 1 mM of total protein concentration; (d) Size distribution of the oligomers at the critical concentration, $(K_A \cdot K_I)^{-1}$, obtained under each experimental condition.
