# Supplementary material for: Characterization of Oligomers of Heterogeneous Size as Precursors of Amyloid Fibril Nucleation of an SH3 Domain: An Experimental Kinetics Study
Source: PLoS One. 2012 Nov 27;7(11):e49690. doi: 10.1371/journal.pone.0049690 (PMC3507826; doi:10.1371/journal.pone.0049690)
Supplement: Text S1 — Model of amyloid nucleation preceded by partial unfolding and oligomerization pre-equilibrium. (PDF) [file pone.0049690.s007.pdf]

Text S1: Model of amyloid nucleation preceded by partial unfolding and oligomerization pre-equilibrium.

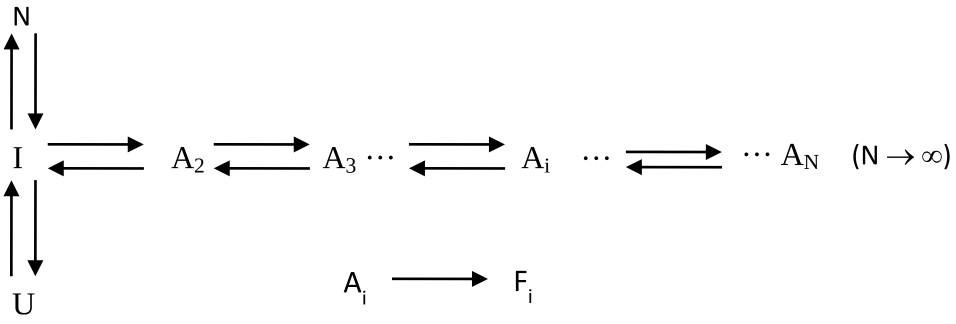

Model description and assumptions:

A partially-unfolded intermediate, I, is in rapid equilibrium with the native, N, and the fully unfolded state, U. The intermediate oligomerizes through a sequential addition of monomers. No limit is imposed to the maximum size of the oligomers, A_i. It is assumed that the conformational equilibria of the monomer species and the oligomerization are fast enough to be pre-equilibrated during the whole process. Amyloid fibril nuclei, F_i, are then formed by a conformational change of the polypeptide chains within the oligomers. Any oligomer can undergo this conformational change and the first order rate constant for this process is equal for all oligomers. We assumed that the formation of amyloid nuclei is essentially irreversible and that further fibril growth by monomer addition does not significantly occur during the early stages of the kinetics, as discussed below.

Mathematical development

The equilibrium constants for the conformational unfolding of the monomer are:

$$K_U = \frac{[U]}{[N]} \qquad K_I = \frac{[I]}{[N]} \qquad (1)$$

And the total concentration of protein monomers at the beginning of the aggregation process:

$$C_0 = [N] + [U] + [I] + \sum_{i=2}^{N} i[A_i] \qquad (2)$$

The fraction of protein in each state is given by:

$$x_N = \frac{[N]}{C_0} \qquad x_I = \frac{[I]}{C_0} \qquad x_U = \frac{[U]}{C_0} \qquad x_{Ai} = \frac{i \cdot [A_i]}{C_0} \qquad (3)$$

Assuming that all equilibrium constants of oligomerization are equal:

$$K_A = \frac{[A_2]}{[I][I]} = \frac{[A_3]}{[A_2][I]} = \frac{[A_i]}{[A_{i-1}][I]} \qquad (4)$$

Using this equation the concentration of any oligomer of size *i* can be related to that of the monomeric intermediate as:

$$[A_i] = K_A [A_{i-1}][I] = K_A^2 [A_{i-2}][I]^2 = \ldots = K_A^{i-1}[I]^i \qquad (5)$$

And the concentration of protein monomers in the intermediate state and in any of the oligomers is given by:

$$[I] + \sum_{i=2}^{\infty} i[A_i] = \sum_{i=1}^{\infty} i[A_i] = \sum_{i=1}^{\infty} i K_A^{i-1}[I]^i \qquad (6)$$

Where $A_1 = I$ in this series. This series is convergent to:

$$\sum_{i=1}^{\infty} i K_A^{i-1}[I]^i = K_A^{-1} \sum_{i=1}^{\infty} i (K_A[I])^i = \frac{K_A^{-1}(K_A[I])}{(1 - K_A[I])^2} \qquad (7)$$

Since for $x < 1$:

$$\sum_{i=1}^{\infty} i \cdot x^i = \frac{x}{(1-x)^2}$$

And using the equilibrium constants K_I and K_U:

$$C_0 = [N](1 + K_U) + \frac{[I]}{(1 - K_A[I])^2} \tag{8}$$

$$1 = x_N(1 + K_U) + \frac{K_I x_N}{(1 - K_A C_0 K_I x_N)^2} = Ax_N + \frac{K_I x_N}{(1 - Bx_N)^2} \tag{9}$$

Where:

$$B = K_A C_0 K_I \quad \text{y} \quad A = 1 + K_U$$

Rearranging this equation we obtain a cubic equation in x_N:

$$AB^2 x_N^3 - (B^2 + 2AB)x_N^2 + (A + 2B + K_I)x_N - 1 = 0 \tag{10}$$

Solving for x_N and using the equilibrium constants we can calculate the concentrations of each state at the time zero of the aggregation process.

The rate of conversion of oligomers A_i into amyloid nuclei F_i is given by:

$$\frac{d[F_i]}{dt} = -\frac{d[A_i]}{dt} = k_{F,i}[A_i] \tag{11}$$

Where $k_{F,i}$ is the first-order constant of the conformational conversion. Assuming that $k_{F,i}$ is independent of the oligomer size, the initial rate of conversion of protein monomers into amyloid structure is:

$$r_0 = \left(\frac{dc_F}{dt}\right)_{t\to 0} = -\sum_{i=2}^{\infty} i\left(\frac{d[A_i]}{dt}\right)_{t\to 0} = \sum_{i=2}^{\infty} i k_{F,i}[A_i]_0 = k_F \sum_{i=2}^{\infty} i[A_i]_0 = \frac{k_F}{K_A}\frac{(Bx_N)^2(2 - Bx_N)}{(1 - Bx_N)^2} \tag{12}$$

Since it can be easily shown that:

$$\sum_{i=2}^{\infty} i[A_i] = K_A^{-1}\sum_{i=2}^{\infty} iK_A^i[I]^i = K_A^{-1}\sum_{i=2}^{\infty} i(Bx_N)^i = \frac{1}{K_A}\frac{(Bx_N)^2(2 - Bx_N)}{(1 - Bx_N)^2} \tag{13}$$

Where we have used the following identity:

$$\sum_{i=2}^{\infty} i \cdot x^i = \frac{x^2(2-x)}{(1-x)^2} \qquad (x < 1)$$

The fraction monomers in each oligomer species A_i is given by:

$$x_{A,i} = \frac{i[A_i]}{C_0} = \frac{i(K_A[I])^i}{C_0 K_A} = \frac{i(Bx_N)^i}{C_0 K_A} \qquad (i = 2\ldots\infty) \qquad (14)$$

And the average number of monomers in the oligomeric species can be easily obtained as:

$$\langle A_n \rangle = \frac{\sum_{i=2}^{\infty} i \cdot [A_i]}{\sum_{i=2}^{\infty} [A_i]} = \frac{\sum_{i=2}^{\infty} i \cdot K_A^{i-1} \cdot [I]^i}{\sum_{i=2}^{\infty} K_A^{i-1} \cdot [I]^i} = \frac{\sum_{i=2}^{\infty} i \cdot K_A^{i} \cdot [I]^i}{\sum_{i=2}^{\infty} K_A^{i} \cdot [I]^i} = \frac{\frac{(Bx_N)^2(2-Bx_N)}{(1-Bx_N)^2}}{\frac{(Bx_N)^2}{(1-Bx_N)}} = \frac{(2-Bx_N)}{(1-Bx_N)} \qquad (15)$$

Where we have made use of the above results and of the fact that for x <1:

$$\sum_{i=2}^{\infty} x^i = \frac{x^2}{1-x}$$
