# Supplementary material for: Characterization of Oligomers of Heterogeneous Size as Precursors of Amyloid Fibril Nucleation of an SH3 Domain: An Experimental Kinetics Study
Source: PLoS One. 2012 Nov 27;7(11):e49690. doi: 10.1371/journal.pone.0049690 (PMC3507826; doi:10.1371/journal.pone.0049690)
Supplement: Text S2 — Model simulations. (PDF) [file pone.0049690.s008.pdf]

Text S2: Model simulations.

Using the equations of the model described in Text S2 we have simulated the initial rates of nucleation as a function of the initial protein concentrations.

1. Amyloid nucleation of a stable partially unfolded amyloidogenic intermediate. In this case the I state is the most stable state. This is equivalent to the linear polymerization model of Oosawa and Kasai[1]. To simulate this case we can asume that K_I is large compared to K_U.

   The following data were calculated for $K_I = 100$, $K_U = 0.12$, and different values of K_A. $k_f = 1\ min^{-1}$.

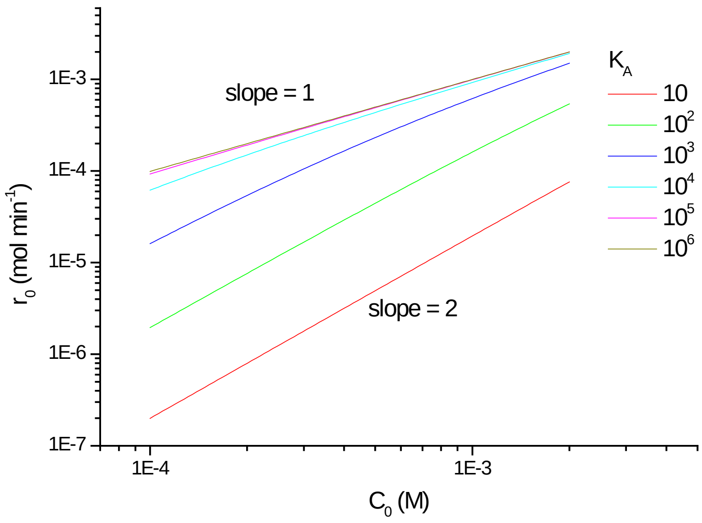

   The slope changes gradually from 2 to 1 as K_A increases. This is logical because for low K_A, the monomer governs the pre-equilibrium but it has to self-associate into dimers to form nuclei. For high values of K_A most of the protein is oligomeric from the beginning of the process and the process appears first order.

2. Amyloid nucleation of a folded protein.

In this case the pre-oligomerization equilibrium is coupled to the folding-unfolding equilibrium. The relative population of I and A_i will be reduced depending on the values of the equilibrium constants K_I and K_U.

a) Effect of K_I and K_A. K_A was varied independently of K_I for K_U = 0.12 and k_f = 1 min^{-1}.

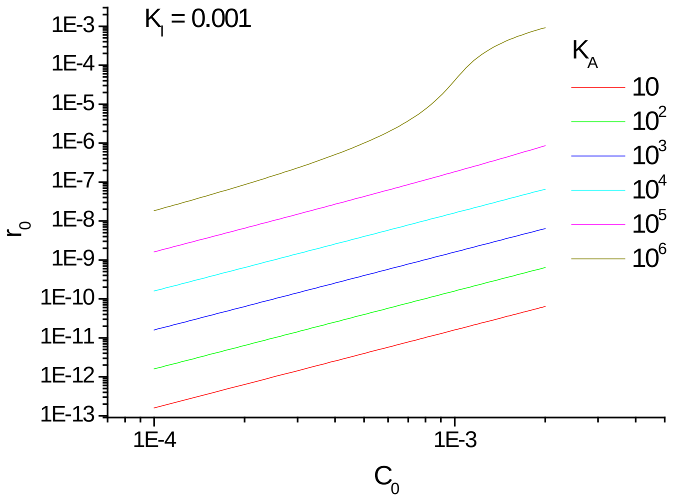

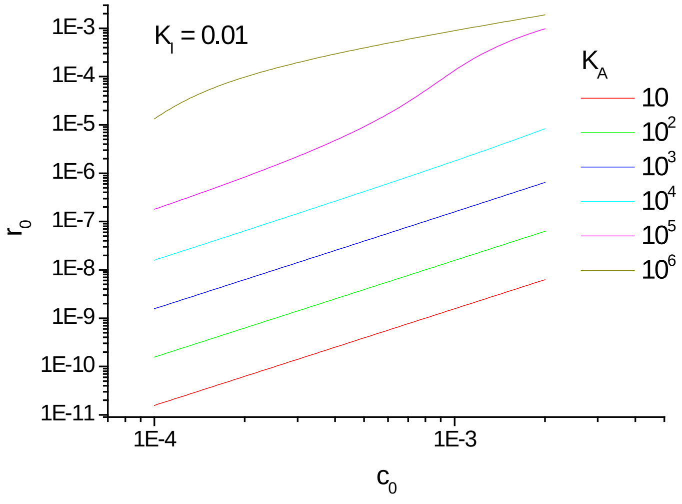

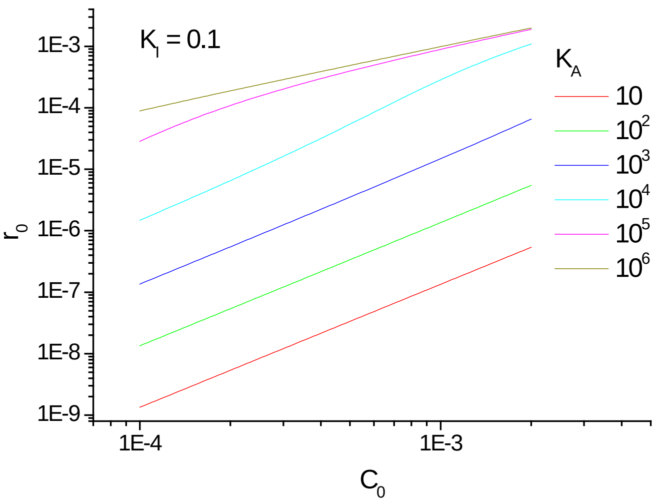

The rate of nucleation depends very strongly on K_A and K_I:

- For $C_0 << (K_AK_I)^{-1}$ the rate of nucleation is very low and scales on the second power of C_0.

- If $C_0 >> (K_AK_I)^{-1}$, the rate tends to a limit imposed by k_F and the nucleation is first order.

- If $C_0 \approx (K_AK_I)^{-1}$, there is a transient increase in the slope of the double logarithmic plot. This is due to an abrupt displacement of the oligomerization equilibrium as C_0 increases. The value $(K_AK_I)^{-1}$ acts as a critical concentration.

b) Next we simulated curves for the same product $K_AK_I = 10^3$ but changing their relative values. K_U and k_F are the same as in a)

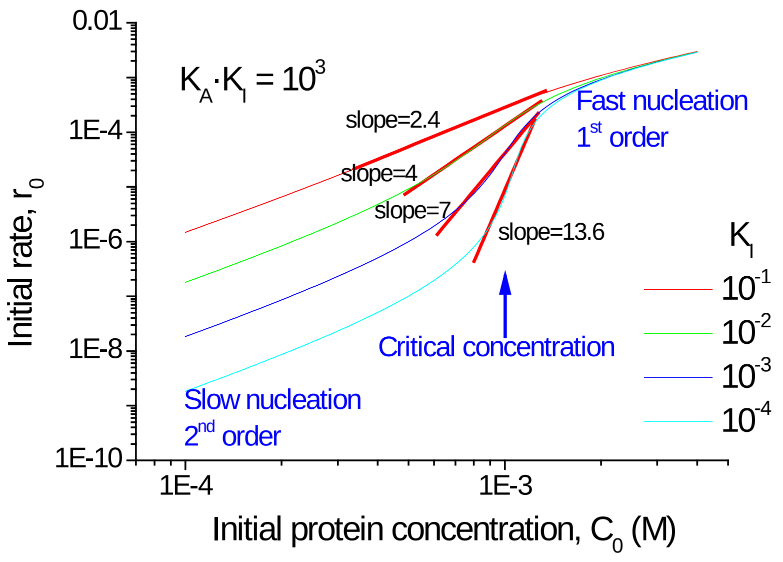

In the región of the critical concentration $C_0 \approx (K_A K_I)^{-1}$ there is a transient slope increase. The slope is higher for higher K_A and lower K_I values.

c) Effect of the global unfolding equilibrium constant K_U:

Simulations were made varying K_U and the resulting values of the unfolding Gibbs energy, ΔG_U. Values of K_A and K_I are as in b)

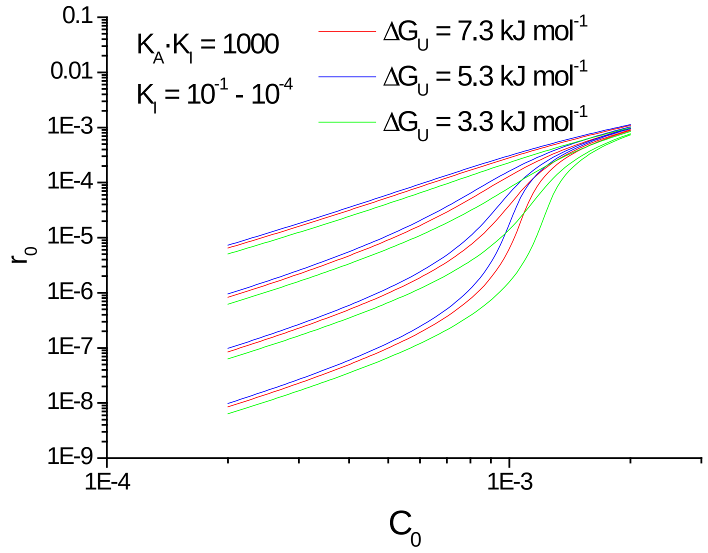

The unfolding Gibbs energy does not have a strong effect on the initial rates because it has a relatively low effect on the population of the amyloidogenic intermediate.

(1) Oosawa, F.; Kasai, M. Theory of Linear and Helical Aggregations of Macromolecules. *J. Mol. Biol.* **1962**, *4*, 10.
