# Supplementary material for: Characterization of Oligomers of Heterogeneous Size as Precursors of Amyloid Fibril Nucleation of an SH3 Domain: An Experimental Kinetics Study
Source: PLoS One. 2012 Nov 27;7(11):e49690. doi: 10.1371/journal.pone.0049690 (PMC3507826; doi:10.1371/journal.pone.0049690)
Supplement: Text S3 — Contribution of fibril elongation by monomer addition to the early kinetics. (PDF) [file pone.0049690.s009.pdf]

Text S3: Contribution of fibril elongation by monomer addition to the early kinetics

We consider now the possibility that monomer addition would also contribute significantly to the initial rate of amyloid growth. During the early stages of the kinetics both the nucleation and elongation can be considered essentially irreversible and the concentrations of the monomeric and oligomeric precursors remain approximately constant and equal to the pre-equilibrated concentrations [2]. With these assumptions, the rate of formation of amyloid nuclei is:

$$\frac{dN_F}{dt} = k_F \sum_{i=2}^{\infty} [A_i] = k_F N_A \qquad (16)$$

$$N_F \approx k_F N_A t \qquad (17)$$

Where N_A and N_F are respectively the number concentrations of oligomer and amyloid particles at a particular time. The rate of aggregation at early times is given by:

$$r = k_F C_A + k_E [I] N_F = k_F C_A + k_F k_E N_A [I] t \qquad (18)$$

Here k_E is the elongation rate constant. This equation implies that if monomer addition was important the the initial slope of the aggregation kinetics would increase and the kinetics would have an upwards curvature.

The relative contribution of monomer addition to the overall rate is:

$$\frac{r_E}{r_F} = \frac{k_E N_A [I] t}{C_A} = \frac{k_E [I] t}{\langle A_n \rangle} \qquad (19)$$

The following simulations show that an increase in slope with time during the early kinetics due to amyloid growth by monomer addition can only be significant for high k_E. On the other hand, formation of oligomers is favored by high protein concentration, decreasing the concentration of intermediate and reducing the contribution of monomer addition.

In these simulations we used $K_A = 2.5 \times 10^5$, K_I ranging between 10^{-3} and 10^{-1}, and k_E = 10^3 and 10^4 M^{-1} min^{-1}.

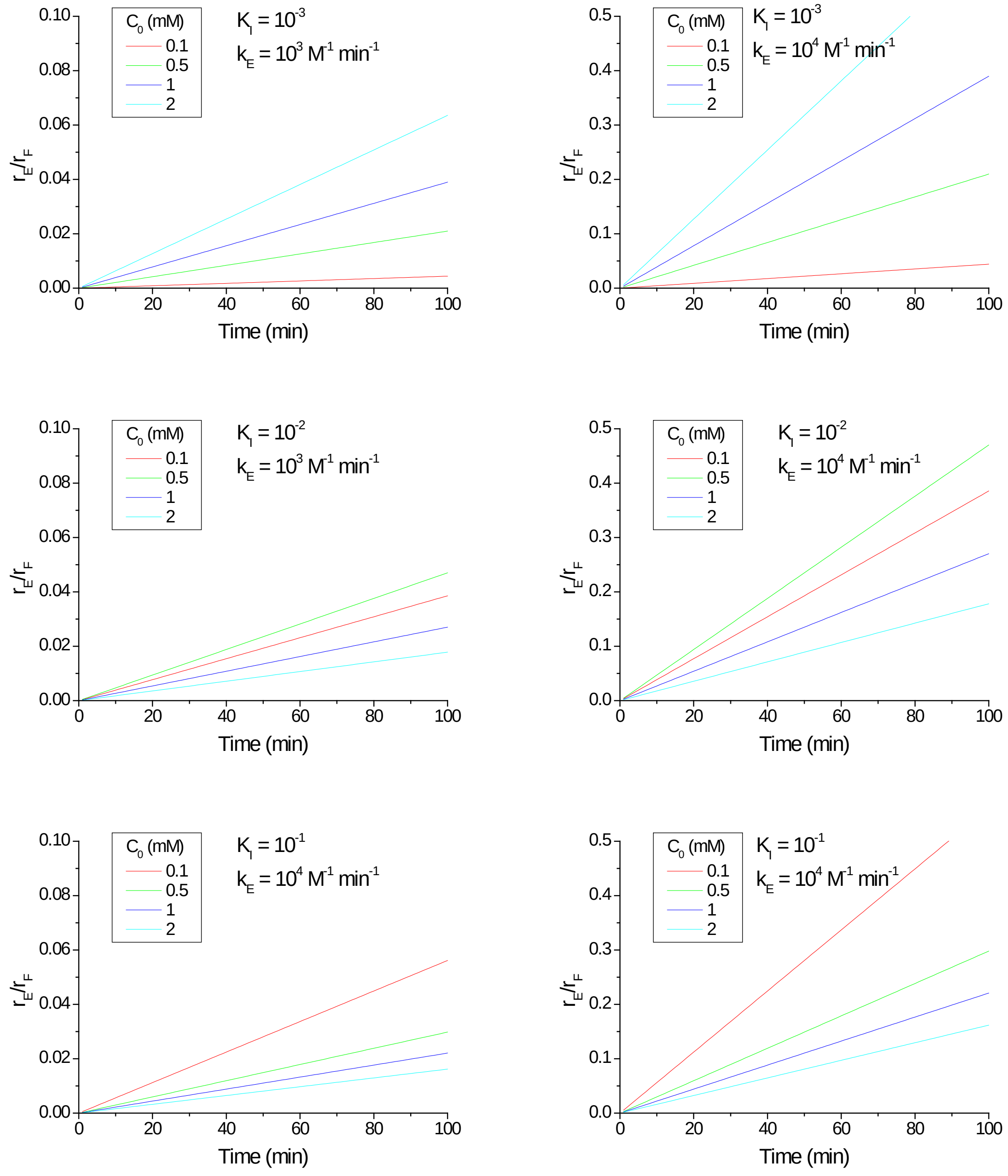

These simulations show that while under most conditions the initial aggregation rates are representative of the formation of nuclei, if the elongation rate constant is high, the contribution of monomer addition can be significant even at short times of aggregation.

This problem could be alleviated by using an appropriate extrapolation of the initial rates at time zero.

(2) Vitalis, A.; Pappu, R. V. Assessing the contribution of heterogeneous distributions of oligomers to aggregation mechanisms of polyglutamine peptides. *Biophys Chem* **2011**, *159*, 14.
